# Optimal 16S rRNA gene amplicon sequencing analysis for oral microbiota to avoid the potential bias introduced by trimming length, primer, and database

**DOI:** 10.1128/spectrum.03512-23

**Published:** 2024-10-22

**Authors:** Takahiko Nagai, Takahiko Shiba, Keiji Komatsu, Takayasu Watanabe, Takashi Nemoto, Shogo Maekawa, Ryota Kobayashi, Shunsuke Matsumura, Yujin Ohsugi, Sayaka Katagiri, Yasuo Takeuchi, Takanori Iwata

**Affiliations:** 1 Department of Periodontology, Graduate School of Medical and Dental Sciences, Tokyo Medical and Dental University, Tokyo, Japan; 2 Department of Lifetime Oral Health Care Sciences, Tokyo Medical and Dental University, Tokyo, Japan; 3 Department of Chemistry, Nihon University School of Dentistry, Tokyo, Japan; The Ohio State University College of Dentistry, Columbus, Ohio, USA

**Keywords:** 16S rRNA gene sequencing, oral microbiota, human oral microbiome database, Greengenes2, dental calculus

## Abstract

16S rRNA gene amplicon sequencing analysis is used to investigate bacterial communities; however, the estimated bacterial composition can differ from the original due to experimental and analytical biases. Therefore, this study determines the optimal conditions to minimize the potential biases in 16S rRNA gene amplicon sequencing analysis using different DNA samples, trimming lengths, primers, and databases. The results of the mock1 community with 250 and 300 bp paired-end (PE), comprising 15 bacteria from various environments, showed the highest similarity between the theoretical value and the data using the Greengenes2 and the V3 region at the genus level and V5–V6 at the species level. In the 300 bp PE sequencing analysis of the mock2 community, comprising six major oral bacteria, the data using the V3–V4, V4, and V5–V6 regions with the SILVA, Greengenes2, and the Human Oral Microbiome Database (HOMD) showed the highest similarity to the theoretical values at the genus level. At the species level, the data using the V3–V4 and V4 regions with Greengenes2 and the data using the V1–V2 with HOMD exhibited the highest alignment with the theoretical values. In the species analysis of the dental calculus samples with 300 bp PE, the Shannon index value was higher with the V1–V2 region and HOMD than with others. Our results suggest that the optimal conditions for oral microbiome analysis are the combinations of 300 bp PE and the V3–V4 or V4 region with the Greengenes2 and the V1–V2 with HOMD.

**IMPORTANCE:**

The 16S rRNA gene amplicon sequencing analysis is frequently used in oral microbiome research. However, this method can have biases that distort the experimental data, which depend on the methodological steps, including sequencing length, trimming length, selected amplification regions, and referenced databases. In this study, a combination of 300 bp PE and the primer targeting the V3–V4 or V4 regions with the Greengenes2 and the V1–V2 region with the HOMD was the most bias-minimizing condition for oral microbiota analysis. In addition, this is the first report of such analyses in modern Japanese dental calculus. The methods used in this study will aid in setting appropriate conditions for sequence analysis of microbiota obtained not only from the oral cavity but also from any environment.

## INTRODUCTION

The bacterial composition of the oral cavity comprises more than 700 bacterial species (1). Periodontal disease is caused by the dysbiosis of this complex microbiome, particularly in dental plaque (2–5), the estimated prevalence of periodontitis was nearly 60% from 2011 to 2020, and higher than that from 1999 to 2010, with its severe stage affecting approximately 24% (6). Periodontitis reportedly exacerbates type 2 diabetes, cardiovascular disease, preterm low birth weight, non-alcoholic fatty liver disease, obesity, and gut inflammation *in vivo* (7–14). Oral bacteria affect the pathophysiology of systemic diseases through the inflammatory response induced by the oral infection or the ectopic accumulation of oral microorganisms and/or their components in other organs. Dental plaques contain numerous oral bacteria and are mineralized to form dental calculus (15, 16). Dental calculus to build up around the gingival margin will lead further to a scaffold for plaque accumulation, thereby promoting the proliferation of periodontal pathogens and exacerbating periodontitis. Therefore, in addition to removing plaque and dental calculus, determining the bacterial composition is important for understanding the pathogenic factors in the diseases caused by dysbiosis, such as periodontitis. 16S rRNA gene amplicon sequencing analysis, an analytical method used to clarify the diversity and complexity of bacterial communities (17), enables cost-effective and easy-to-use for analyzing bacterial composition (18). However, 16S rRNA gene amplicon analysis may introduce a bias between the true and real data in the results obtained from bacterial communities, which is influenced by each methodological step (19).

The steps in which the bias arises for 16S rRNA gene amplicon sequencing analysis are sampling, DNA extraction method, primer selection, sequencing method, software for analysis, and database selection (18). In a study using a mock oral community, the primers targeting the V3–V4 and V4–V5 regions reportedly yielded higher result reproducibility (20). However, in studies using mock communities, the V1–V3 region was the most accurate for the compositional evaluation of oral microbiota using saliva samples with SILVA (21) and using subgingival plaque samples with Greengenes and HOMD (22). A previous study using Quantitative Insights Into Microbial Ecology version 2 (QIIME2) and a primer targeting the V3–V4 region demonstrated that paired-end reads could be merged when low-quality regions of the sequenced read were trimmed, and the trimming length was optimized to obtain appropriate overlapping regions; this improved the accuracy of the analysis (23, 24). However, the recommended trimming length of the sequenced reads for various primers in QIIME2 has not been investigated, except for the primer targeting the V3–V4 region. Abellan-Schneyder et al. (18) reported that the SILVA ribosomal RNA database (SILVA) and the Ribosomal Database Project displayed more accurate taxonomic classification of gut microbiota in human stool samples than the Greengenes, the genomic-based 16S rRNA Database, or the All-Species Living Tree database (18). Although Greengenes2, which combines the metagenomic and 16S rRNA databases into a single database, has recently been released, its utility for oral samples has not been evaluated (25). The Human Oral Microbiome Database (HOMD; www.homd.org) mainly consists of data from 16S rRNA gene amplicon sequencing analysis of oral bacteria and is frequently utilized for analyzing oral microbiota (26). The combination of the HOMD and the V1–V2 primer is useful for analyzing oral samples in comparison with the V4 and V3–V4 primers since the V1–V2 primer better identified most *Streptococci* at the species level (17). Although *Streptococci* are predominant in the oral cavity, the high diversity of the oral microbiota generates controversy regarding the reliability of species-level results and the conditions most suitable for analyzing these bacteria using the 16S rRNA gene sequencing. In addition, although the sequencing length of 300 bp yields higher confident results than that from the 250 bp when using the primer targeting the V3–V4 region (27), a comparison between the analysis accuracy of the 250 and 300 bp length has not been reported when using primers targeting other regions and a mock community. Therefore, determining the optimal conditions for 16S rRNA gene amplicon sequencing analysis in the oral cavity is necessary for understanding microbiota.

**Fig 1:**
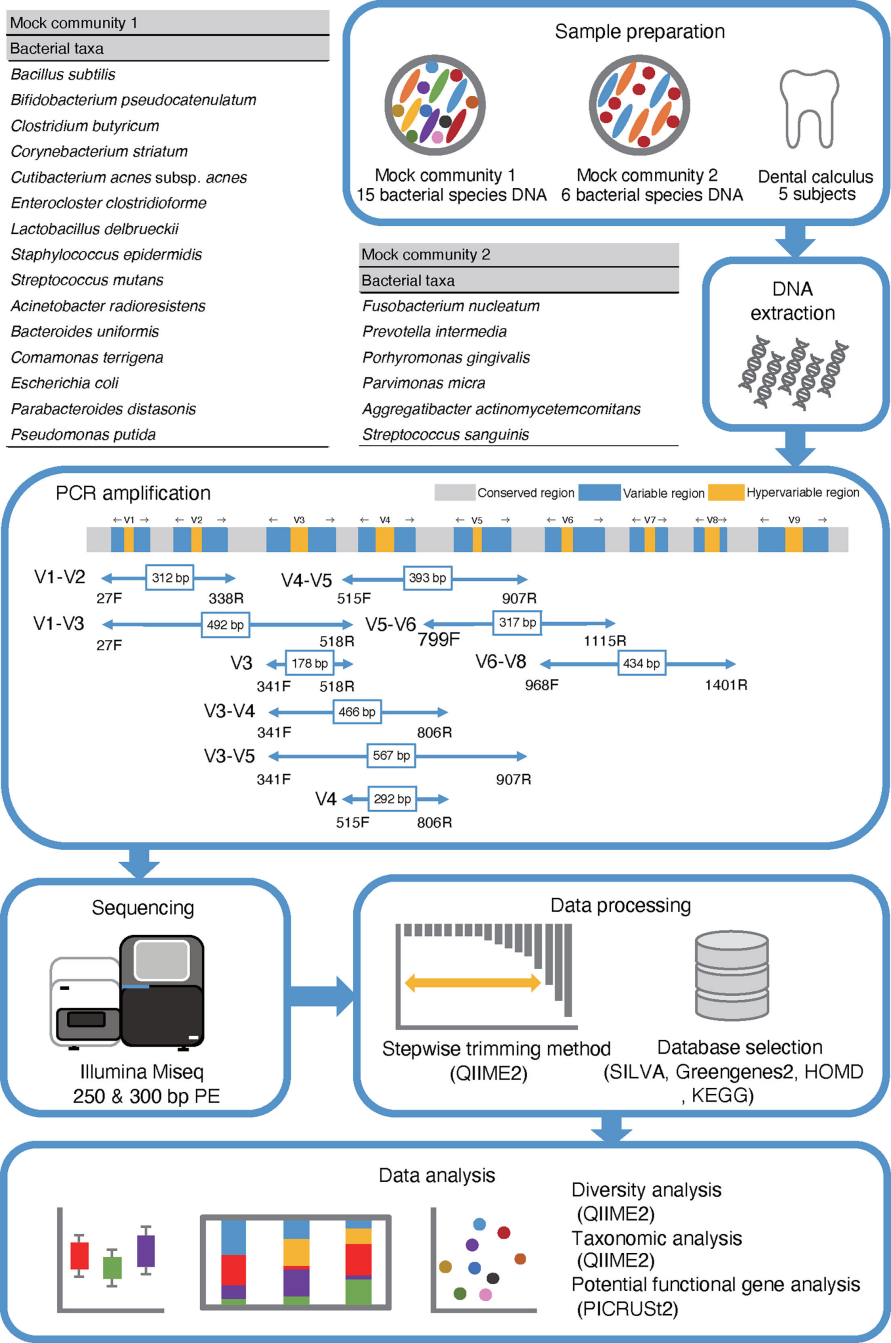
Overview of the experiments using two mock communities and the dental calculus samples, nine types of primer, sequencing lengths, software, databases, and data analyses. The mock1 community consisted of 15 bacterial taxa associated with various environments, the mock2 community consisted of six taxa associated with the oral cavity, and dental calculus samples were prepared. DNA was extracted, targeted hypervariable regions in the 16S rRNA gene were amplified with nine types of primers, library preparation and sequencing of DNA amplicons with 250 and 300 bp PE were performed, and the sequenced data were processed using the QIIME2 pipeline and the SILVA, Greengenes2, and HOMD to analyze the diversity and taxonomy. Potential functional genes were analyzed using KEGG and PICRUSt2.

In this study, we comprehensively investigated each methodological step to determine the optimal condition for oral microbiota in 16S rRNA gene amplicon sequencing analysis using two mock communities and dental calculus samples, two lengths of sequencing read, and stepwise trimming of the paired-end sequenced reads, nine types of primers targeting different hypervariable regions, and three reference databases (Fig. 1).

## RESULTS

### Analysis of optimal trimming and merging lengths for QIIME2

**Fig 2:**
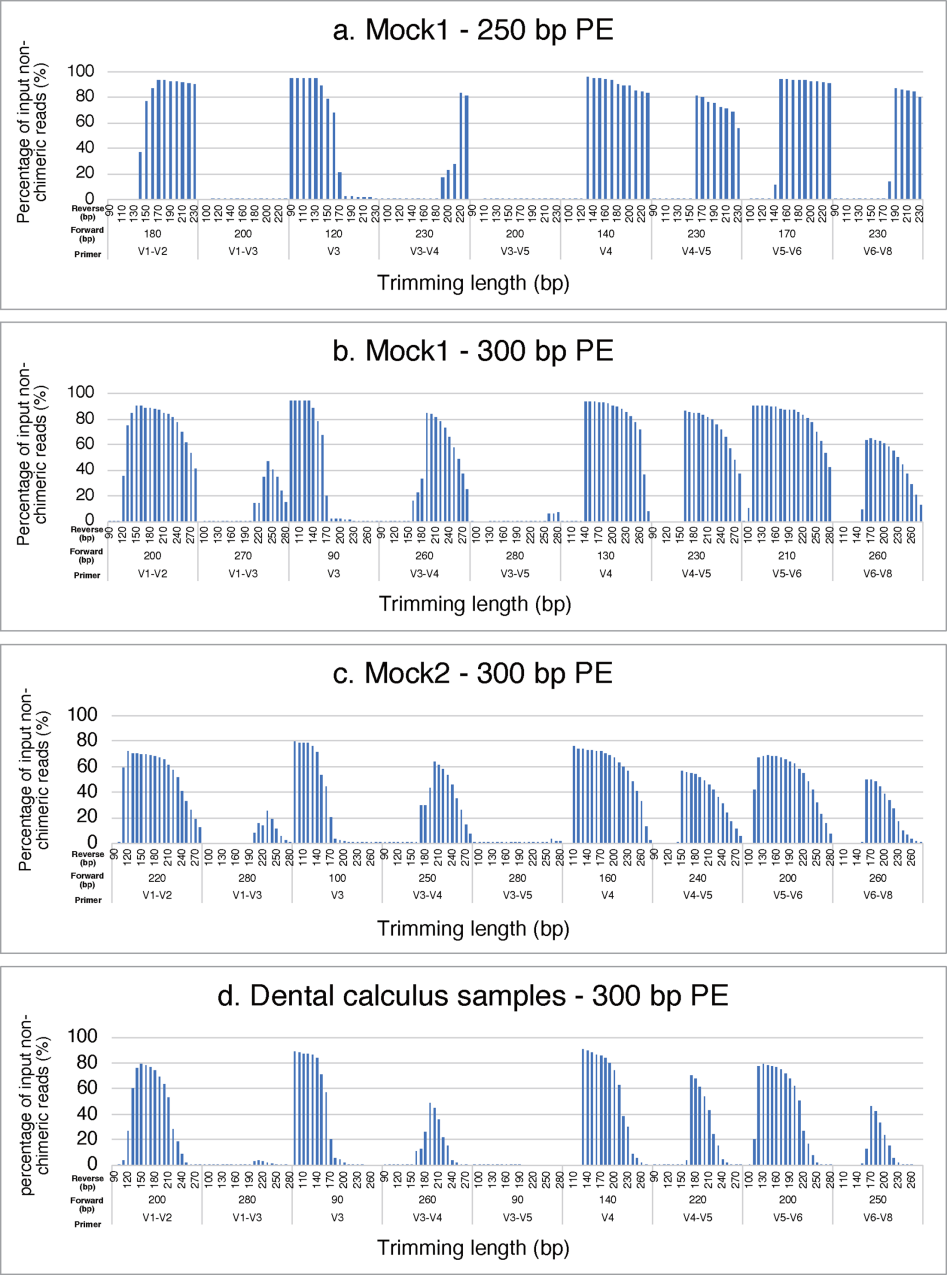
Investigation of the appropriate trimming lengths in mock1, mock2, and dental calculus samples. The vertical axis is the average survival of the non-chimeric reads obtained in DADA2 of QIIME2; the horizontal axis is the trimming length of the forward and reverse reads. The length of the forward read indicates the maximum survival of the non-chimeric reads and the stepwise trimming length of the reverse read in each primer. Results are shown for the 250 bp PE of mock1 (**a**), the 300 bp PE of mock1 (**b**), the 300 bp PE of mock2 (**c**), and the 300 bp PE of the dental calculus sample (**d**).

The mean percentage of the survival rate of the sequenced reads, which were filtered, merged, and denoised in QIIME2, varied based on the sample, primer type, and trimming length of the sequenced read (Fig. 2; Tables S1 to S4; Fig. S1). The survival rate of the sequenced reads was maximized with specific trimming lengths of the forward and reverse reads, which differed for each primer even with the same sample and sequencing length. Conversely, the survival rate of sequenced reads decreased with the under- and over-trimming length. The highest survival rates were 95.83% for V4 in the 250 bp paired-end (PE) of mock1, 94.64% for V3 in the 300 bp PE of mock1, 79.23% for V3 in the 300 bp PE of mock2, and 90.36% for V4 in the 300 bp PE of the dental calculus sample (Table 1; Tables S1 to S4; Fig. S1). The top two primers with the highest survival rates were V3 and V4, while the bottom two were V1–V3 and V3–V5 in all samples. The data from V1–V3 and V3–V5 in the 250 bp PE analysis of mock1 and V3–V5 in the 300 bp PE analysis of the dental calculus samples were excluded from further analysis since the non-chimeric read survival rates were below 10%. It was confirmed that the remaining data showed sufficient survival reads for subsequent analyses using rarefaction curves (Fig. S2). In addition, the length of the overlapped regions varied based on the sample, primer type, and sequencing length, ranging from 68–78 bp for V1–V2, 0–57 bp for V1–V3, 38–87 bp for V3, 23 bp for V3–V4, 0–32 bp for V3–V5, 19 bp for V4, 38 bp for V4–V5, 40–60 bp for V5–V6, and 22–32 bp for V6–V8 (Table 1).

**TABLE 1:** Summary of the trimmed length for maximizing the survival rate of the non-chimeric read^*a*^^,^^*b*^

Primer	Forward (bp)	Reverse (bp)	Overlap (bp)	Filtered (%)	Merged (%)	Non-chimeric (%)
**a** Mock1—250 bp PE						
V1-V2	180	170	78	96.17	95.08	93.09
V1-V3	200	220	Less than 0	89.33	0.03	0.03
V3	120	110	88	98.52	95.23	95.05
V3-V4	230	220	23	84.89	83.69	83.23
V3-V5	200	210	Less than 0	67.49	0.02	0.02
V4	140	130	19	98.71	97.21	95.83
V4-V5	230	160	38	86.77	84.21	81.08
V5-V6	170	150	40	98.60	96.37	93.87
V6-V8	230	190	22	94.05	91.28	86.70
**b** Mock1—300 bp PE						
V1-V2	200	150	78	94.59	93.17	90.63
V1-V3	270	240	57	58.75	48.39	47.00
V3	90	90	38	97.91	95.11	94.64
V3-V4	260	190	23	87.30	86.05	85.10
V3-V5	280	280	32	15.86	7.43	7.21
V4	130	140	19	97.84	96.87	93.72
V4-V5	230	160	38	94.09	91.70	86.25
V5-V6	210	110	40	96.09	94.41	90.74
V6-V8	260	170	32	85.46	82.01	64.74
**c** Mock2—300 bp PE						
V1-V2	220	120	68	84.13	80.16	71.86
V1-V3	280	230	57	30.23	28.00	25.29
V3	100	90	48	88.42	84.85	79.23
V3-V4	250	200	23	69.45	66.77	64.08
V3-V5	280	260	12	7.94	3.36	3.27
V4	160	110	19	79.54	78.22	76.07
V4-V5	240	150	38	62.86	60.42	56.45
V5-V6	200	140	60	84.39	80.72	68.91
V6-V8	260	170	32	67.63	61.56	50.24
**d** Dental calculus samples—300 bp PE						
V1-V2	200	150	78	86.90	83.90	78.93
V1-V3	280	210	37	13.32	4.02	3.65
V3	90	90	38	93.85	92.04	88.58
V3-V4	260	190	23	52.22	51.26	48.92
V3-V5	90	130	Less than 0	77.42	0.08	0.08
V4	140	130	19	94.87	93.92	90.36
V4-V5	220	170	38	78.40	75.64	70.51
V5-V6	200	130	50	90.67	86.88	79.04
V6-V8	250	170	22	57.82	53.43	46.04

^*a*^ The list of trimmed lengths of the forward and reverse reads that maximize the survival rate of the non-chimeric reads. Results are shown for the 250 bp PE of mock1 (a), the 300 bp PE of mock1 (b), the 300 bp PE of mock2 (c), and the 300 bp PE of the dental calculus sample (d).

^*b*^ The results are shown for the 250 bp PE of mock1 (a), the 300 bp PE of mock1 (b), the 300 bp PE of mock2 (c), and the 300 bp PE of the dental calculus sample (d).

### Diversity, phylogenetic, and similarity analysis of the 250 bp PE of mock1

**Fig 3:**
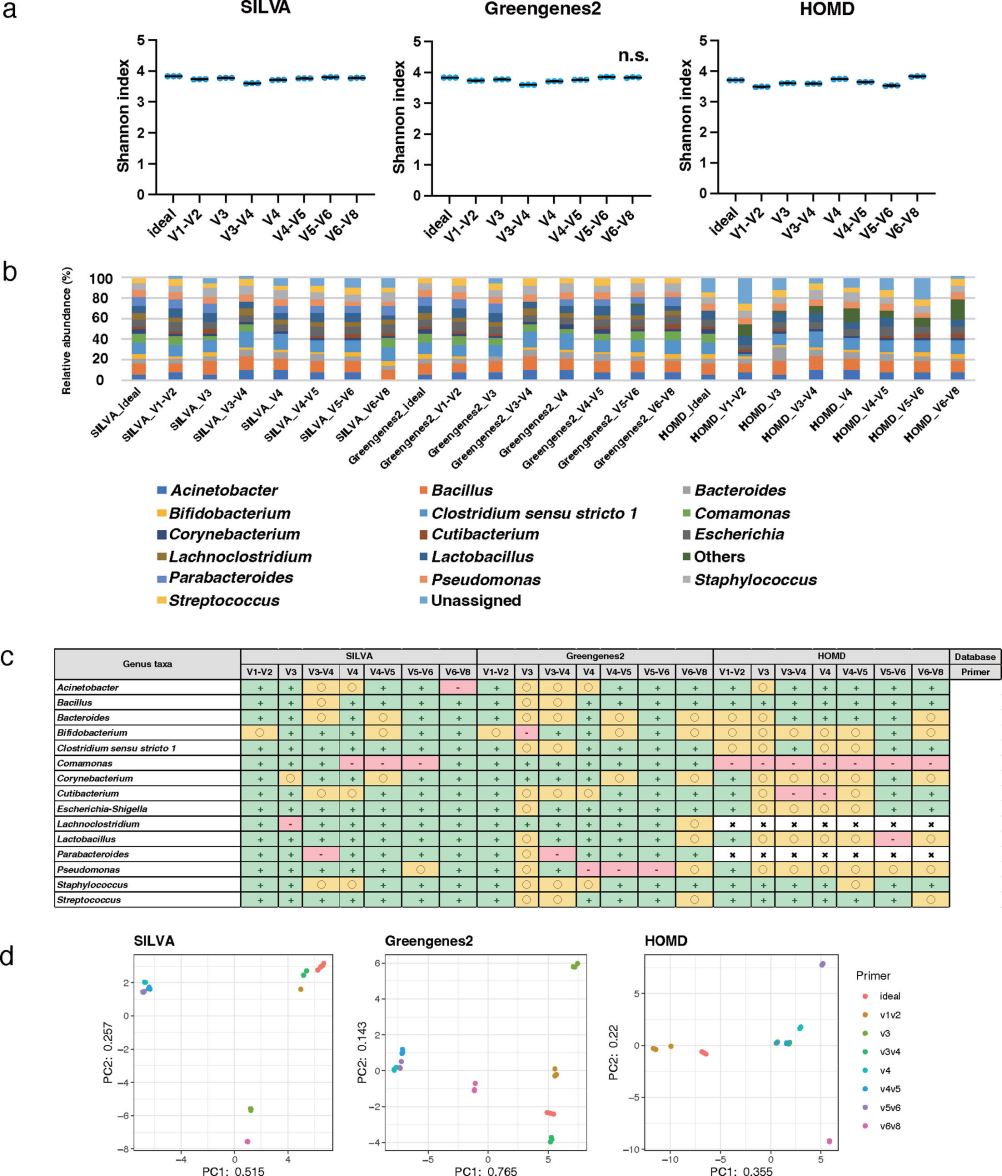
Genus-level analyses of data obtained from mock1 with 250 bp PE. The results of alpha diversity analysis using the Shannon index at the genus level for each database are shown in scattered dot plots. Each value was averaged from three replicates. Data showing no significant difference from the ideal data are indicated as "n.s." (**A**). Results of the phylogenetic analysis at the genus level for each database are shown in a bar graph based on relative bacterial abundance at 100% conversion. Each value was averaged from three replicates (**b**). The degree of analytical accuracy for each bacterial taxa comprising the mock communities was calculated based on CLR value. Each value was averaged from three replicates. The CLR value that was within ±5% of error from the theoretical value, was symbolized as “+,” within ±5% to ±25% as “○,” and less than −25% or more than 25% as “-.” Bacteria not registered in the database were symbolized as “×” (**c**). The results of the similarity of the bacterial composition at the genus level for each database are shown in the PCA, and each sample had three replicates, based on the CLR value (**d**).

The genus-level analysis results of mock1, using the 250 bp PE sequencing length, were evaluated (Tables S5 to S7). All 15 genera in mock1 were registered in SILVA and Greengenes2, and 13 of these 15 genera were included in HOMD. The Shannon index values of the samples ranged from 3.6 to 3.8 for SILVA, 3.6 to 3.8 for Greengenes2, and 3.5 to 3.8 for HOMD; the value based on SILVA and Greengenes2 was a slightly higher than that of HOMD (Fig. 3a). For the Shannon index, there was no significant difference between the ideal data and the data of the V6–V8 with Greengenes2; however, significant differences were observed between the ideal data and the data of the other groups with all databases (*P* < 0.01). In addition, the relative abundance ratios differed for each primer and database (Fig. 3b). The centered log ratio (CLR) values for *Streptococcus*, which is generally predominant in the oral microbiota, showed relatively accurate results, within ±5% error, compared with the ideal conditions when using the various primers targeting specific regions including the V1–V2, V4, V4–V5, and V5–V6 for each database. However, the data of *Streptococcus* obtained using the V3, V3–V4, and V6–V8 primers with Greengenes2 and the V6–V8 with HOMD showed relatively inaccurate results within ±5% to ±25% error (Fig. 3c). The principal component analysis (PCA) showed that the variation between theoretical and sample values differed among primers and databases (Fig. 3d). The bacterial composition obtained using the combination of the V3 primer and Greengenes2 closely approached the theoretical value based on Spearman’s rank correlation coefficient (r 0.898, *P*  <  0.01; Table 2a).

**TABLE 2:** Results of the similarity analysis using Spearman’s correlation coefficient^*a*^

	a						b						c						d						E						F					
Sample	Mock1						Mock1						Mock1						Mock1						Mock2						Mock2					
Read length	250 bp PE						250 bp PE						300 bp PE						300 bp PE						300 bp PE						300 bp PE					
Classification	Genus						Species						Genus						Species						Genus						Species					
Database	SILVA		Greengenes2		HOMD		SILVA		Greengenes2		HOMD		SILVA		Greengenes2		HOMD		SILVA		Greengenes2		HOMD		SILVA		Greengenes2		HOMD		SILVA		Greengenes2		HOMD	
Number of registered bacteria	15/15		15/15		13/15		15/15		15/15		7/15		15/15		15/15		13/15		15/15		15/15		7/15		6/6		6/6		6/6		6/6		6/6		6/6	
Value	r	*P*	r	*P*	r	*P*	r	*P*	r	*P*	r	*P*	r	*P*	r	*P*	r	*P*	r	*P*	r	*P*	r	*P*	r	*P*	r	*P*	r	*P*	r	*P*	r	*P*	r	*P*
ideal	1.000	**	1.000	**	1.000	**	1.000	**	1.000	**	1.000	**	1.000	**	1.000	**	1.000	**	1.000	**	1.000	**	1.000	**	1.000	**	1.000	**	1.000	**	1.000	**	1.000	**	1.000	**
V1-V2	0.881	**	0.881	**	0.600	*	−0.102	-	0.554	*	0.890	**	0.881	**	0.881	**	0.600	*	−0.107	-	0.531	*	0.888	**	0.829	*	0.829	*	0.893	**	0.036	-	0.829	*	0.893	**
V1-V3	-	-	-	-	-	-	-	-	-	-	-	-	0.708	**	0.672	**	0.755	**	0.044	-	0.272	-	0.640	*	0.600	-	0.600	-	0.750	-	0.214	-	0.600	-	0.750	-
V3	0.880	**	0.898	**	0.711	**	−0.260	-	0.035	-	0.322	-	0.888	**	0.907	**	0.693	**	−0.152	-	0.233	-	0.254	-	0.829	*	0.829	*	0.893	**	−0.519	-	0.829	*	−0.519	-
V3-V4	0.744	**	0.744	**	0.589	*	0.263	-	0.453	-	0.467	-	0.862	**	0.869	**	0.795	**	0.345	-	0.655	**	0.444	-	>0.999	**	>0.999	**	>0.999	**	−0.519	-	>0.999	**	−0.342	-
V4	0.580	*	0.518	*	0.735	**	−0.047	-	0.354	-	0.453	-	0.612	*	0.580	*	0.742	**	0.014	-	0.509	*	0.585	*	>0.999	**	>0.999	**	>0.999	**	−0.519	-	>0.999	**	−0.342	-
V4-V5	0.585	*	0.710	**	0.755	**	−0.047	-	0.464	-	0.604	*	0.609	*	0.677	**	0.781	**	0.014	-	0.528	*	0.585	*	0.829	*	0.829	*	0.893	**	−0.519	-	0.829	*	−0.342	-
V5-V6	0.491	-	0.780	**	0.481	-	−0.395	-	0.868	**	0.696	**	0.482	-	0.780	**	0.481	-	−0.276	-	0.851	**	0.672	*	>0.999	**	>0.999	**	>0.999	**	−0.519	-	0.143	-	−0.342	-
V6-V8	0.552	*	0.746	**	0.285	-	0.079	-	0.528	*	0.563	*	0.576	*	0.762	**	0.280	-	0.177	-	0.652	**	0.538	-	0.943	**	0.943	**	0.964	**	−0.519	-	0.086	-	−0.342	-

^*a*^ The lists of Spearman's correlation coefficient values and P-value; the p-value < 0.05, is shown as "＊", and the p-value < 0.01 is shown as "＊＊", of 250 bp PE in mock1 at the genus- level (a), the values of 250 bp PE in mock1 at the species- level (b), the values of 300 bp PE in mock1 at the genus- level (c), the values of 300 bp PE in mock1 at the species- level (d), and the values of 300 bp PE in mock2 at the genus- level, the values of 300 bp PE in mock2 at the species- level.

**Fig 4:**
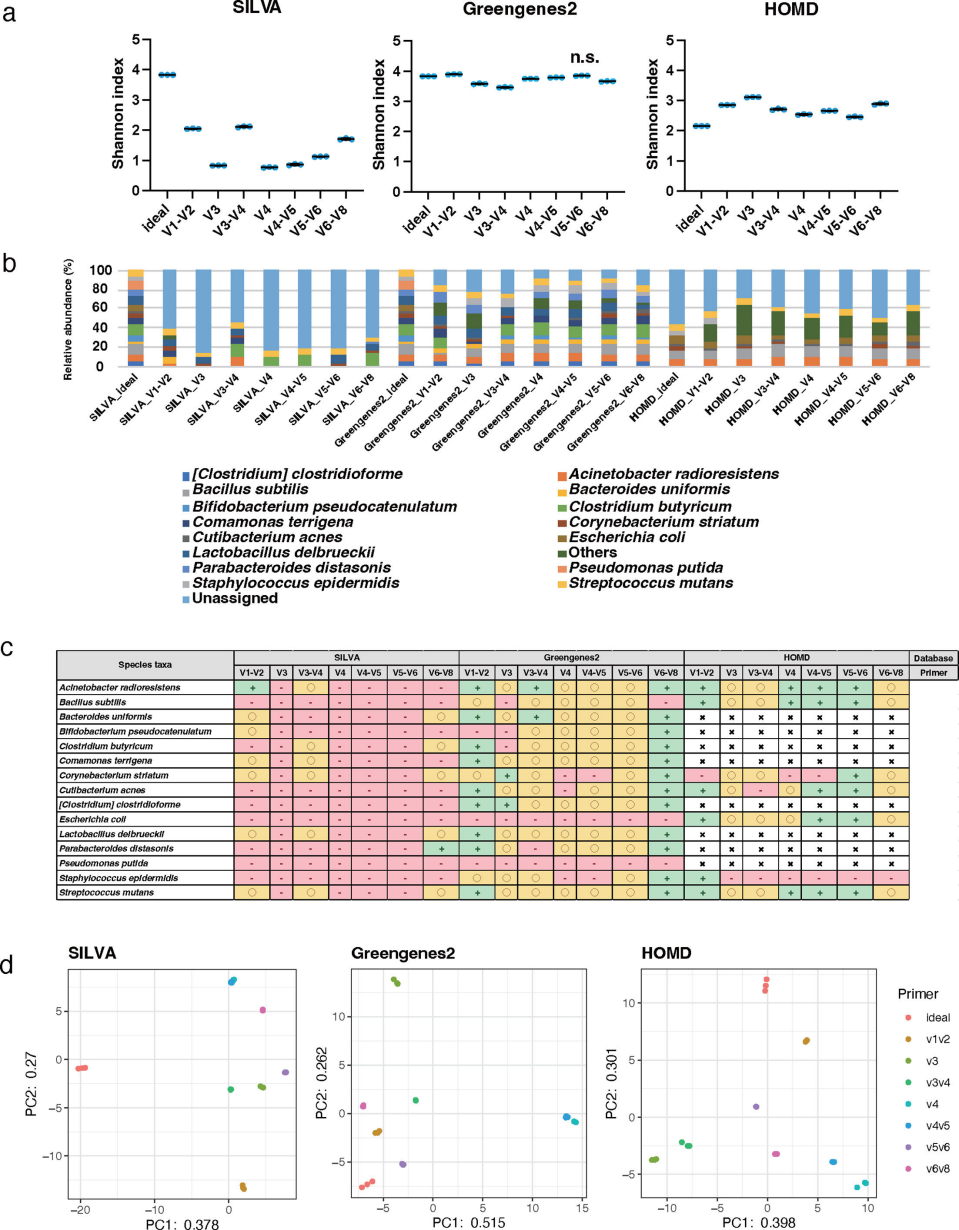
Species-level analyses of data obtained from mock1 with 250 bp PE. The scattered dot plots were prepared in the same manner as described in Fig. 2a. Data showing no significant difference from the ideal data are indicated as "n.s." (**a**). A bar graph was constructed in the same manner in the same manner as described in Fig. 2b (**b**). A table is presented in the same manner as described in Fig. 2c (**c**). PCA was conducted in the same manner as described in Fig. 2d (**d**).

Among the 15 species, 15, 15, and 7 were registered with SILVA, Greengenes2, and HOMD, respectively. The Shannon index values at the species level ranged from 0.8 to 2.1 for SILVA, 3.5 to 3.9 for Greengenes2, and 2.5 to 3.1 for HOMD; the value based on Greengenes2 was relatively higher than those of SILVA and HOMD (Fig. 4a). For the Shannon index, there was no significant difference between the ideal data and the data of the V5–V6 with Greengenes2; however, significant differences were observed between the ideal data and the data of the other groups with all databases (*P* < 0.01). The proportion of data classified as “Unassigned” increased more at the species level than at the genus level and accounted for a large percentage in almost all the analyzed data (Fig. 4b). The CLR value of *Streptococcus mutans*, which is mainly related to dental caries, showed relatively accurate results, within ±5% error, compared with the ideal conditions when using the V1–V2 and V6–V8 primers with the Greengenes2 and the V1–V2, V4, V4–V5, and V6–V8 with the HOMD. Conversely, the data obtained using the V3, V4, V4–V5, and V5–V6 primers with the SILVA showed relatively inaccurate results with over ±25% error (Fig. 4c). PCA showed that the variation between theoretical and real values at the species level exceeded that at the genus level for each primer (Fig. 4d; Table 2a and b). The data obtained using the combination of the V5–V6 primer and Greengenes2 was the closest approach to the theoretical value among the combinations at the species level based on Spearman’s rank correlation coefficient (r 0.868, *P* <  0.01; Table 2b).

### Diversity, phylogenetic, and similarity analysis of the 300 bp PE of mock1

**Fig 5:**
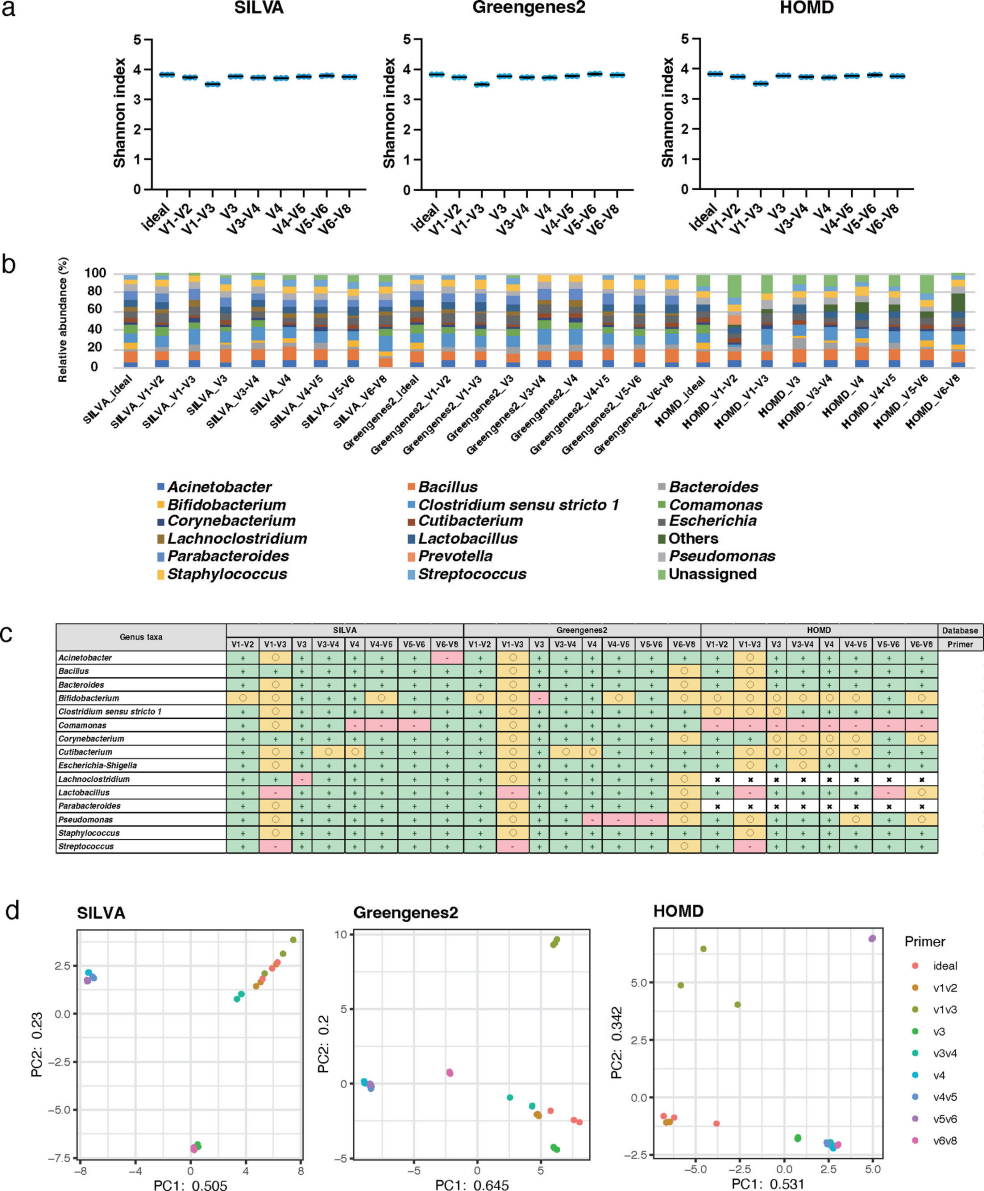
Genus-level analyses of data obtained from mock1 with 300 bp PE. The scattered dot plots were prepared in the same manner as described in Fig. 2a (**a**). A bar graph was constructed in the same manner as described in Fig. 2b (**b**). A table is presented in the same manner as described in Fig. 2c (**c**). PCA was conducted in the same manner as described in Fig. 2d (**d**).

The genus-level analysis of mock1 using 300 bp PE sequencing length yielded Shannon index values of 3.5–3.8 for SILVA, 3.5–3.8 for Greengenes2, and 3.3–3.8 for HOMD (Fig. 5a). For the Shannon index, significant differences were observed between all groups and the ideal data with all databases (*P* < 0.01). The discrepancy in the bacterial composition was observed between the theoretical values and those obtained with each primer (Fig. 5b; Tables S8 to S10). The CLR value of *Streptococcus* showed results within ±5% error when using the V1–V2, V3, V3–V4, V4, V4–V5, and V5–V6 primers with all databases; however, the data obtained using the V1–V3 primer with all databases showed relatively inaccurate results with over ±25% error compared with ideal conditions (Fig. 5c). The data obtained for the CLR value of Parabacteroides, which is mainly related to the gut microbiome, showed relatively accurate results within ±5% error as compared with ideal conditions when using all primers except for the V1–V3 primer with the SILVA and the V1–V3 and V6–V8 with Greengenes2 (Fig. 5c). The data variability based on the database is shown as a PCA plot (Fig. 5d). However, despite having the same database, the results showed that the variability lay within the primers. The data obtained using the combination of the V3 primer and Greengenes2 closest approached the theoretical value based on Spearman’s rank correlation coefficient (r 0.907, *P*  <  0.01; Table 2c).

**Fig 6:**
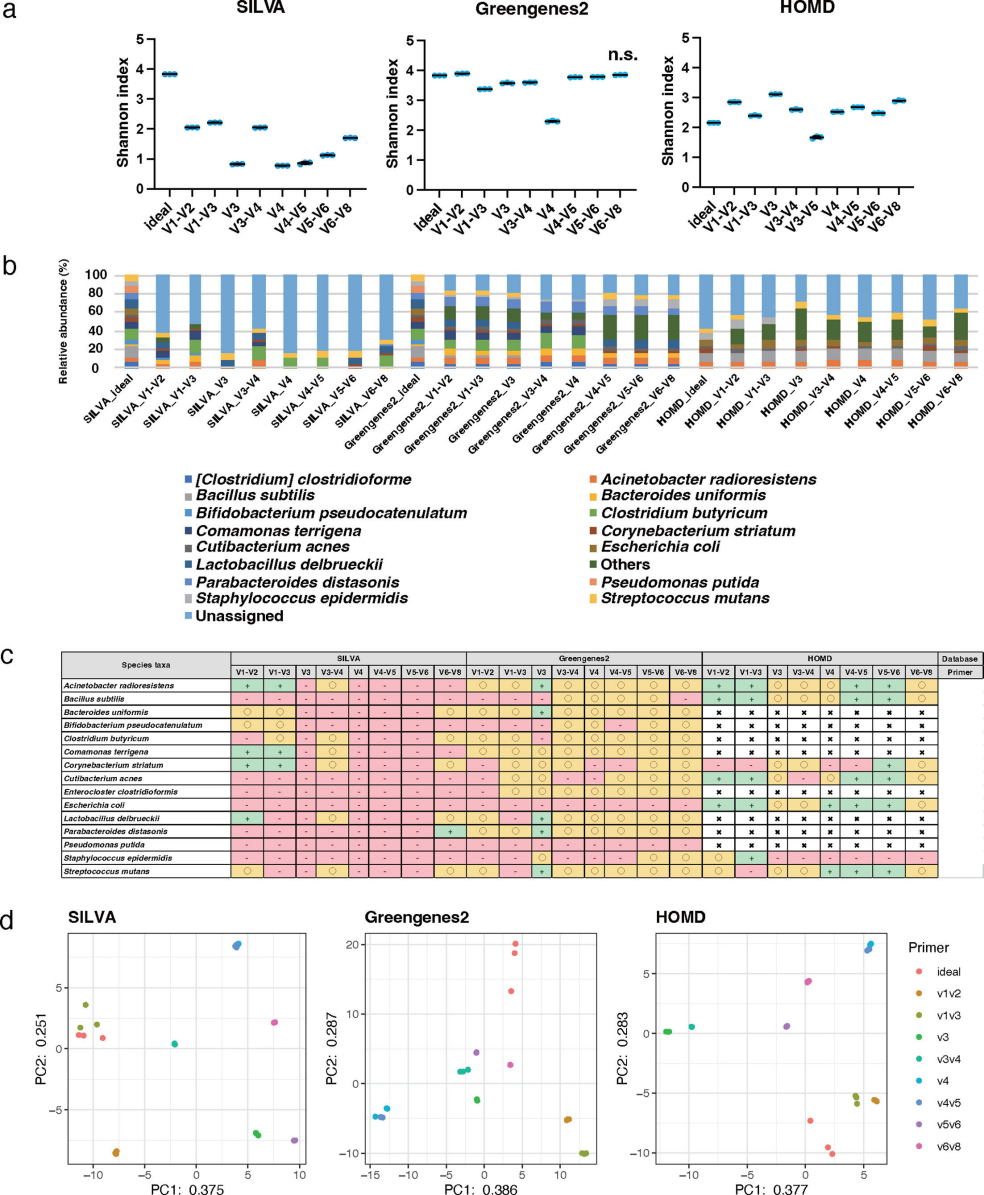
Species-level analyses of data obtained from mock1 with 300 bp PE. The scattered dot plots were prepared in the same manner as described in Fig. 2a. Data showing no significant difference from the ideal data are indicated as "n.s." (**a**). A bar graph was constructed in the same manner as described in Fig. 2b (**b**). A table is presented in the same manner as described in Fig. 2c (**c**). PCA was conducted in the same manner as described in Fig. 2d (**d**).

At the species level, the Shannon index values of each primer ranged from 0.8 to 2.2 for SILVA, 2.3 to 3.9 for Greengenes2, and 1.7 to 3.1 for HOMD; the value based on Greengenes2 was slightly higher than those of SILVA and HOMD (Fig. 6a). For the Shannon index, there was no significant difference between the ideal data and the data of the V6–V8 with Greengenes2; however, significant differences were observed between the ideal data and the data of the other groups with all databases (*P* < 0.01). The bar plot at the species level showed a more remarkable deviation from the theoretical values relative to that observed at the genus level (Fig. 6b). In addition, the proportion of the “Unassigned” data increased at the species level compared with the genus level. The CLR value of *Streptococcus mutans* showed results within ±5% error when using the V3 primer with the Greengenes2 and the V4, V4–V5, and V5–V6 and HOMD; however, the data obtained using the V1–V3 primer with all databases and the V3, V4, V4–V5, and V5–V6 with the SILVA showed relatively inaccurate results with over ±25% error compared with the theoretical value (Fig. 6c). The distances between the theoretical and real values were greater at the species level than genus level (Fig. 6d; Table 2c and d). The data obtained using the combination of the V5–V6 primer and Greengenes2 were the closest approach to the theoretical value based on Spearman’s rank correlation coefficient (r 0.851, *P*  < 0.01; Table 2d).

### Diversity, phylogenetic, and similarity analysis of the 300 bp PE of mock2

**Fig 7:**
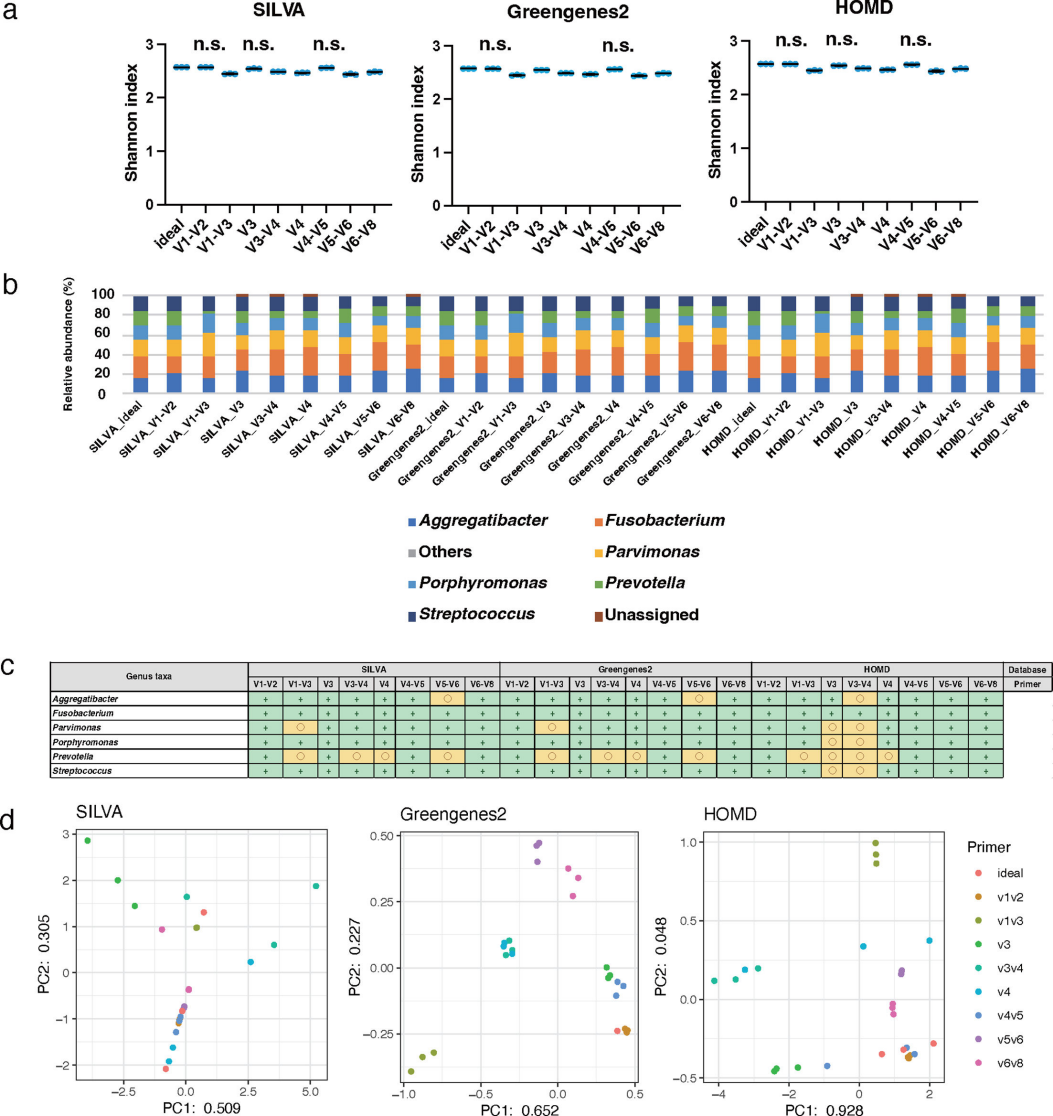
Genus-level analyses of data obtained from mock2 with 300 bp PE. The scattered dot plots were prepared in the same manner as described in Fig. 2a. Data showing no significant difference from the ideal data are indicated as "n.s." (**a**). A bar graph was constructed in the same manner as described in Fig. 2b (**b**). A table is presented in the same manner as described in Fig. 2c (**c**). PCA was conducted in the same manner as described in Fig. 2d (**d**).

The genus-level analysis results of mock2 using 300 bp PE sequencing length were analyzed (Tables S11 to S13). Mock2 was composed of six bacteria associated with the oral microbiome, and all six species were registered in SILVA, Greengenes2, and HOMD. The Shannon index values ranged from 2.4 to 2.6 for SILVA, 2.4 to 2.6 for Greengenes2, and 2.4 to 2.6 for HOMD (Fig. 7a). For the Shannon index, there were no significant differences between the ideal data and the data of the V1–V2 and V4–V5 groups with all databases and V3 with SILVA and HOMD; however, significant differences were observed between the ideal data and the data of the other groups with all databases (*P* < 0.01). Although all six species were detected in all samples, their relative abundance ratios differed from the theoretical composition values at the genus levels (Fig. 7b). The CLR values of all bacterial taxa showed a relatively accurate result within ±25% error compared with the theoretical value when using all primers and databases (Fig. 7c). Even though the database was the same, the variation between theoretical and real values varied based on the primers used (Fig. 7d). The data obtained using the combination of the V3–V4, V4, and V5–V6 primers with Greengenes2 and HOMD approached the theoretical value based on Spearman’s rank correlation coefficient (r > 0.999, *P*  < 0.01; Table 2e).

**Fig 8:**
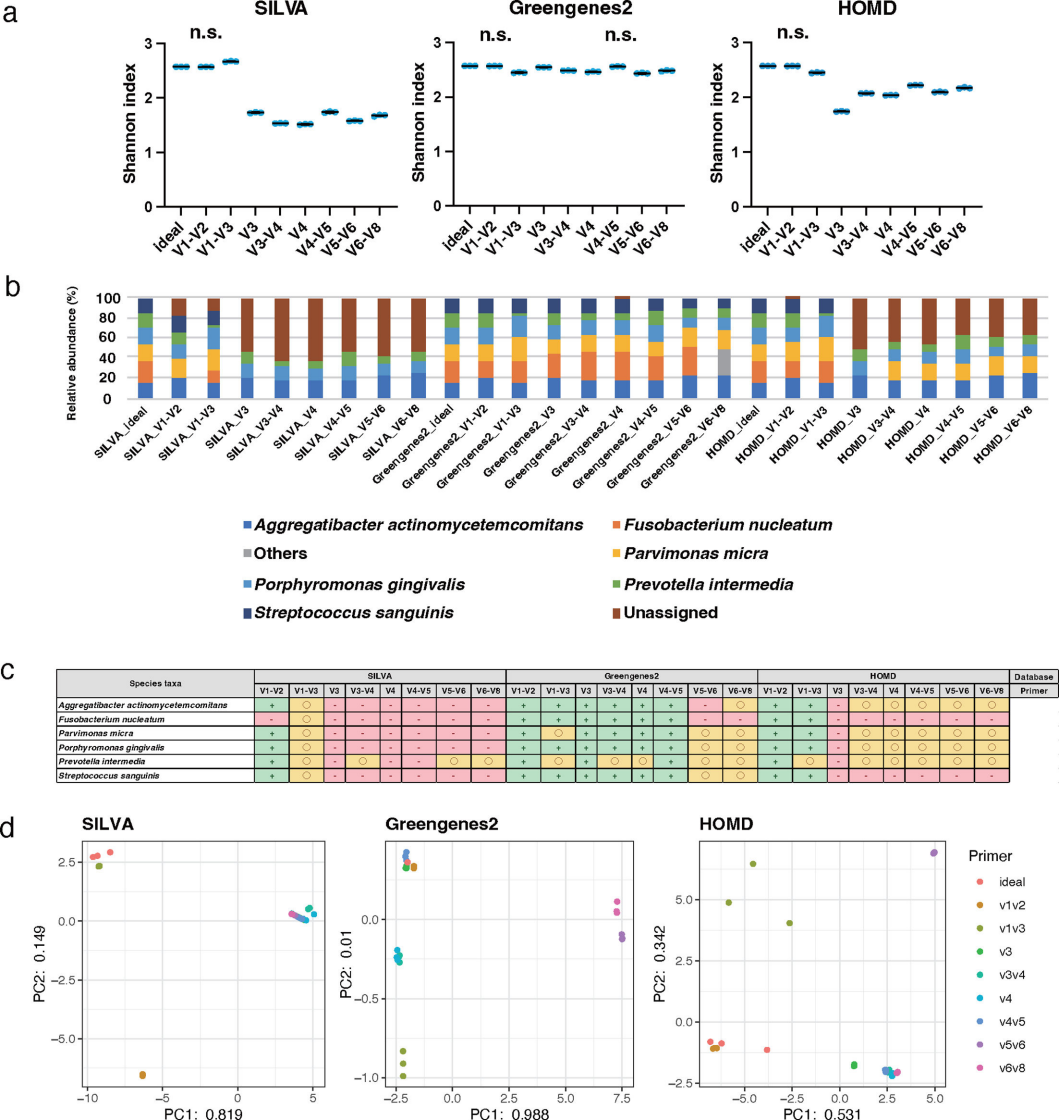
Species-level analyses of data obtained from mock2 with 300 bp PE. The scattered dot plots were prepared in the same manner as described in Fig. 2a. Data showing no significant difference from the ideal data are indicated as "n.s." (**a**). A bar graph was constructed in the same manner as described in Fig. 2b (**b**). A table is presented in the same manner as described in Fig. 2c (**c**). PCA was conducted in the same manner as described in Fig. 2d (**d**).

All six species were registered at the species level in SILVA, Greengenes2, and HOMD. The Shannon index values ranged from 1.5 to 2.7 for SILVA, 2.4 to 2.6 for Greengenes2, and 1.7 to 2.6 for HOMD, with relatively higher values for Greengenes2 than those for SILVA and HOMD (Fig. 8a). For the Shannon index, no significant differences were observed between the ideal data and the data of the V1–V2 group in all databases and V4-V5 with Greengenes2; however, significant differences between the ideal data and the data of the other groups in all databases were observed (*P* < 0.01). All six species were detected in V1–V3 of SILVA, all primers of Greengenes2, and V1–V2 and V1–V3 of HOMD; only parts of the bacteria were encountered in other conditions (Fig. 8b). The CLR values of all bacterial taxa showed a relatively accurate result with within ±5% error compared with the theoretical value when using the V1–V2, V3, and V4–V5 primers with the Greengenes2 and the V1–V2 with the HOMD. On the other hand, the data obtained using the V1–V2 primer with the SILVA showed a relatively accurate result within ±5% of error compared with the theoretical value in all bacterial taxa except for *Fusobacterium nucleatum* (Fig. 8c). The distances between the theoretical and real values were greater for all data at the species level than at the genus level (Fig. 8d; Table 2e and f). The data obtained using the combination of the V3–V4 and V4 primers with Greengenes2 and the V1–V2 with HOMD closely approached the theoretical value based on Spearman’s rank correlation coefficient (r > 0.999, *P*  < 0.01 and r 0.810, *P*  < 0.01, respectively; Table 2f).

### Diversity and phylogenetics of the 300 bp PE of the dental calculus samples

**Fig 9:**
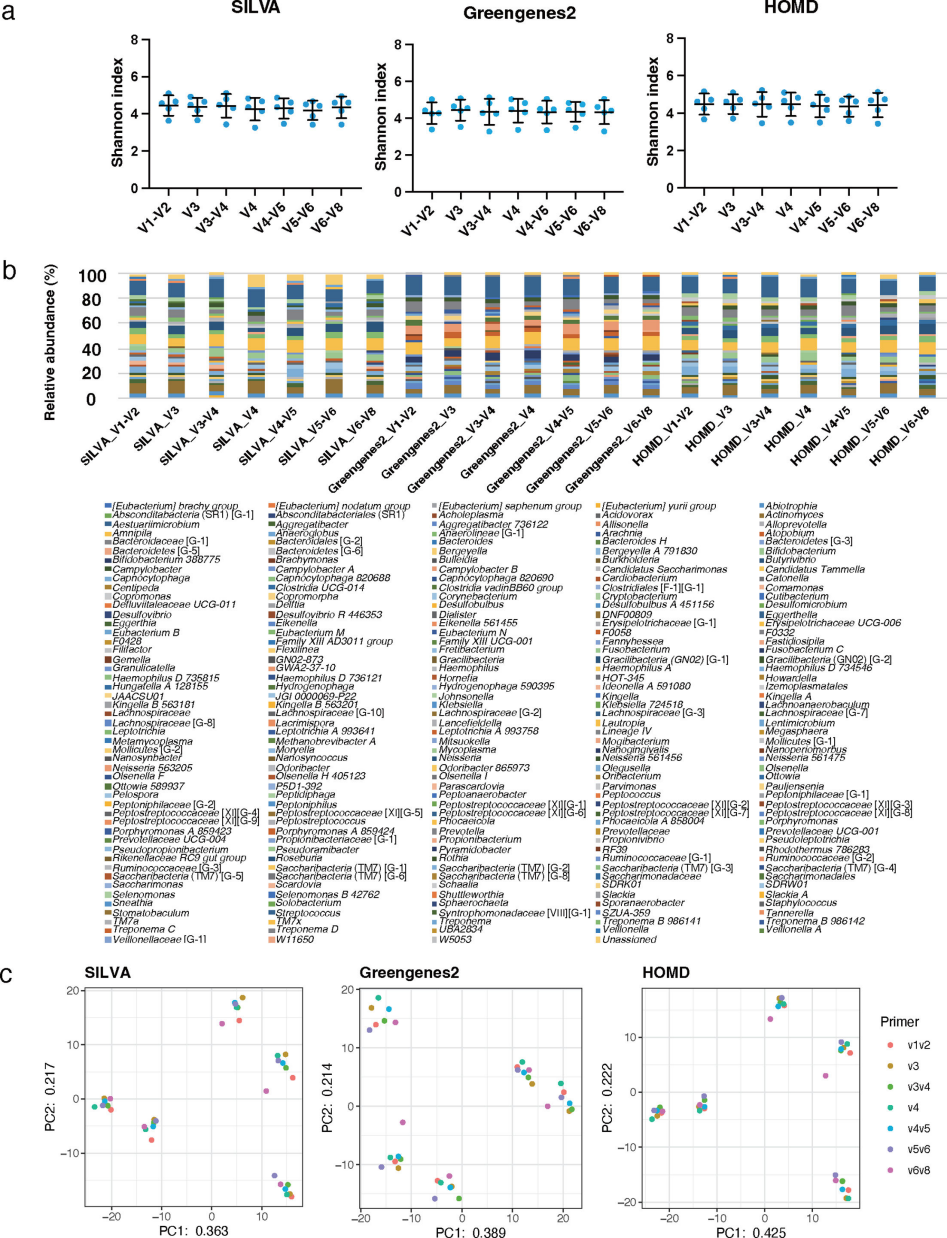
Genus-level analyses of data obtained from dental calculus samples with 300 bp PE. The scattered dot plots were prepared in the same manner as described in Fig. 2a (**a**). A bar graph was constructed in the same manner as described in Fig. 2b (**b**). PCA was conducted in the same manner as described in Fig. 2d (**c**).

The supragingival dental calculus was collected from patients undergoing maintenance and analyzed using 300 bp PE sequencing length (Tables S14 to S16). At the genus level, the Shannon index values of the samples were 4.2–4.3 for SILVA, 4.3–4.4 for Greengenes2, and 4.4–4.5 for HOMD (Fig. 9a). The bar plot results indicated a relatively similar number of bacteria at the genus level; however, the combinations of databases and primers influenced a relative bacterial component (Fig. 9b). The common and dominant bacteria in all data were *Actinomyces* with an average relative abundance of 6.47%, *Fusobacterium* with 4.68%, *Lautropia* with 9.35%, *Neisseria* with 6.59%, *Prevotella* with 5.56%, and *Streptococcus* with 12.91%. The top 10 bacterial genera based on their CLR values in the dental calculus samples using V1-V2 primer and Greengenes2 and V3-V4 and HOMD were shown (Fig. S3). The top 10 genera of both combinations included the above taxa, but their order was different. The variation between PCA plots of each sample differed in all databases, and the characteristics of the sample itself have more impact on the degree of aggregation of each PCA plot than that of the primers (Fig. 9c).

**Fig 10:**
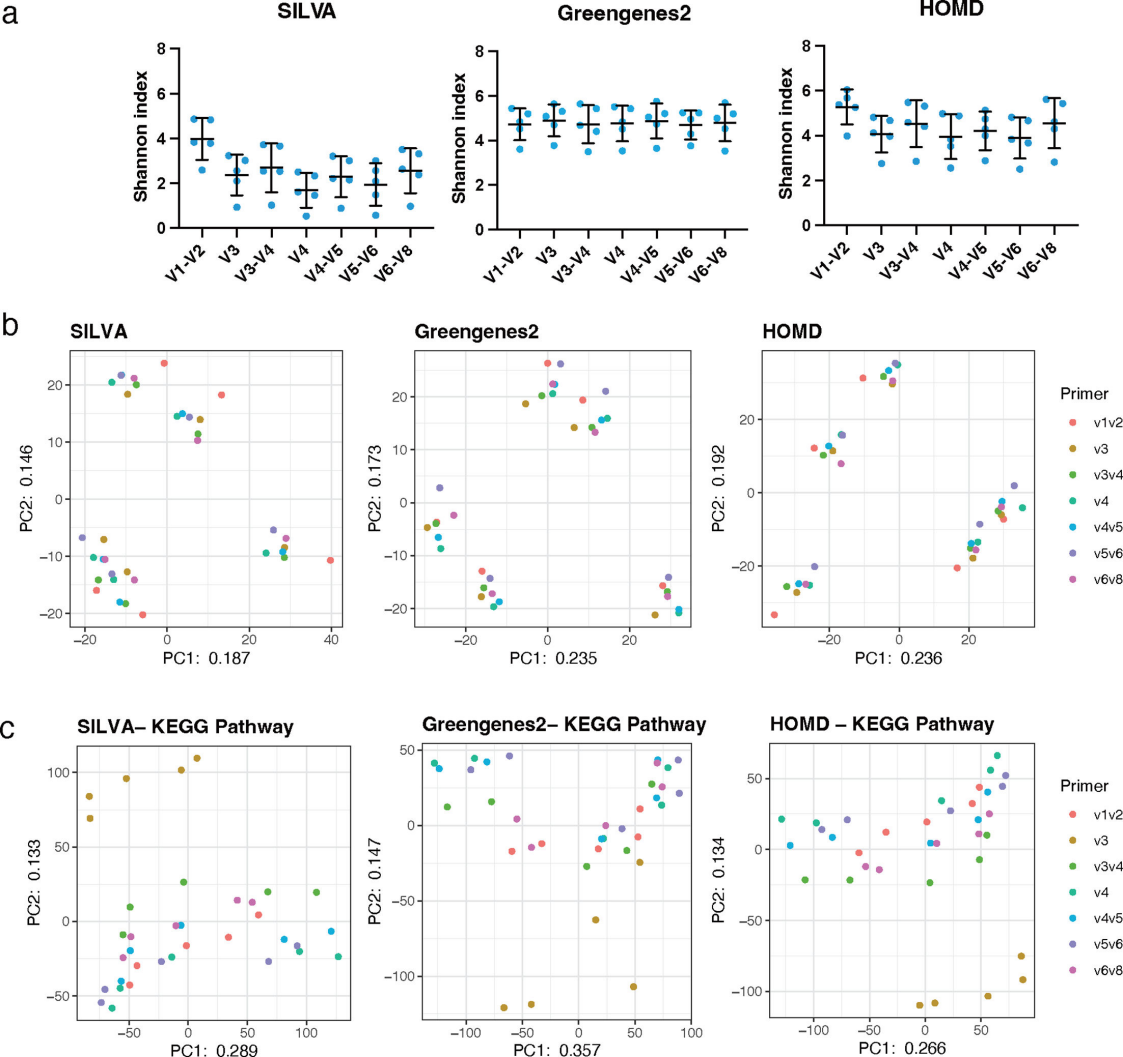
Species-level analyses of data obtained using dental calculus samples with 300 bp PE. The scattered dot plots were prepared in the same manner as described in Fig. 2a (**a**). The PCA was conducted in the same manner as described in Fig. 2d (**b**). The results of the functional similarity for the KEGG pathway are shown in the PCA, based on CLR converted from the raw bacterial abundance using each database (**c**).

At the species-level analysis, the Shannon index values ranged from 1.7 to 4.0 for SILVA, 4.7 to 4.9 for Greengenes2, and 4.0 to 5.3 for HOMD, with relatively higher values for Greengenes2 and HOMD (Fig. 10a). The attributions of the sample itself have more impact on the degree of aggregation of each PCA plot than that of the primers (Fig. 10b). On the other hand, according to the PCA based on the potential functional genes, the influences of the sample itself were smaller than the results of the phylogenetic analysis, and each plot appeared as a single cluster except for the data using V3 primer (Fig. 10c).

## DISCUSSION

The findings of this study suggested the optimal conditions for analyzing oral microbiota using 16S rRNA gene amplicon sequencing (Table 1; Fig. 2; Fig. S1; Tables S1 to S4). The optimal conditions of sequencing length, primer type, trimming length, and database in 16S rRNA gene amplicon analysis differed between the mock1 and mock2 communities. In the mock1 analysis, the primer targeting the V3 region with the Greengenes2 showed the most accurate result at the genus-level analysis for the 250 and 300 bp PE. At the species-level analysis of the mock1, the V5–V6 primer and the Greengenes2 for the 250 and 300 bp PE yielded reliable results (Table 2a to d). In the mock2 analysis, the conditions of 300 bp PE, the primer targeting V3–V4, V4, and V5–V6 regions, and the Greengenes2 and HOMD showed the most accurate result at the genus level. At the species-level analysis for the mock2, the primer targeting the V3–V4 and V4 regions and Greengenes2 and the V1–V2 region and HOMD yielded reliable results (Table 2e and f). These variations in the optimal conditions for the 16S rRNA gene amplicon sequence were influenced by various factors, including primer selection, sequencing length, trimming length, and database.

The requirements for the proper merging of paired-end reads by DADA2 are as follows: (i) the length of the amplified region is shorter than the sequencing read length, (ii) the trimmed site for the forward read is a length whose 25th percentile sequencing quality is close to 20, and (iii) the length of the reverse read is adjusted so that the minimum overlap length is at least 16 bp (24). The minimum required lengths for other primers are as follows: 343 bp for V1–V2, 523 bp for V1–V3, 209 bp for V3, 497 bp for V3–V4, 598 bp for V3–V5, 323 bp for V4, 424 bp for V4–V5, 348 bp for V5–V6, and 465 bp for V6–V8. It is theoretically possible to merge paired-end reads for all nine sets of primers with the 300 bp PE. However, the primers requiring most of the read length, such as the V1–V3 for 523 bp and V3–V5 for 598 bp, cannot obtain sufficient overlapped regions by being trimmed to low-quality regions, which reduces the non-chimeric survival rate of the paired-end reads. Especially, the 300 bp PE is possibly superior to the 250 bp PE in expanding the primer options such as the V1-V3 primer, although there is a limitation for too-long regions, such as the V3–V5 region. Therefore, it is essential to consider the sequencing length to cover the minimum required length, depending on each primer and read quality. It is also important to focus on the analytical accuracy between the 250 bp and 300 bp PEs. A previous study using the primer targeting the V3–V4 region reported that data generated using the 300 bp PE protocol had a longer overlap region, making it superior to the 250 bp PE for generating highly reliable paired-end assemblies (27). Consistent with that report, this study showed that the many primers targeting different regions, including the V3–V4 primer, produced higher accuracy at both the genus and species level with SILVA and Greengenes2 in the mock1 with the 300 bp PE compared with the 250 bp PE. Therefore, we focused on the influence of databases and primers for oral microbiome analysis using the mock2 with 300 bp PE. However, specific combinations, such as the V5–V6 primer with the SILVA at the genus level, showed slightly lower accuracy in the 300 bp PE than in the 250 bp PE. It can be assumed that the 300 bp PE is superior to the 250 bp PE in terms of analytical accuracy, except for the specific combination of primer and database. When using the 250 bp PE, it is important to consider the length of the targeting region and its read quality to retain sufficient nucleotide information covered within the 250 bp PE after amplicon and trimming of reads. The 300 bp PE has the advantage of covering longer reads and handling lower quality reads compared with the 250 bp PE.

The survival rate of non-chimeric reads seems to be related to the length of the amplified region. Shorter regions, such as the V3 and V4, showed higher rates of non-chimeric reads, whereas longer regions, such as the V1–V3, showed lower rates. On the other hand, the analytical accuracy did not necessarily improve in proportion to the higher survival rate of the non-chimeric reads in this study. This suggests that to improve the accuracy of phylogenetic analysis, it is necessary to select primers appropriate for the sample type. Nevertheless, the appropriate trimming length, which maximizes the survival rate of the non-chimeric reads, should be investigated to maintain enough information for analyzing the samples from obtained sequence reads in case an appropriate primer is selected. We manually trimmed the pair-end reads at each length to summarize and present the changes in the survival rates depending on the trimming length. Recently, the FIGARO tool was developed to determine optimal trimming lengths based on the read quality scores (28).

The results’ error compared with the theoretical values was larger at the species level than at genus levels for mock1 and mock2. Previous reports using partial 16S rRNA gene sequences suggested that short reads are less suitable for studying accurate richness estimation and assigning taxa than full-length reads (29, 30) and long-read sequencing provides good resolution for bacterial identification (31). However, in this study, at the species-level analysis of mock1, the Spearman correlation coefficient value showed the highest coefficient value using the primer targeting the V5–V6 region and the Greengenes2, which was 0.868 for the 250 bp PE and 0.851 for the 300 bp PE. At the species-level analysis of mock2 with 300 bp PE, the coefficient value was high, which was obtained using the primer targeting the V3–V4 and V4 regions and Greengenes2 and the V1–V2 regions and HOMD, with >0.999 and 0.893, respectively. These results suggest the specific combination of the primer and database can apply to the species level for a partial 16S rRNA gene amplicon analysis. A possible explanation for this accuracy using Greengenes2 is the development of the database, which has the reference tree unifying the metagenomic and 16S rRNA databases in a consistent, resulting in an improvement in the taxonomic assignment of the partial 16S rRNA gene analysis (25, 32). We also confirmed that the analytical accuracy of the Greengenes was relatively lower than that of Greengenes2 in this study (Table S17). In addition, QIIME2 has recently released a new plugin for metagenomic mapping using Kraken2 as an alpha release (https://cap-lab.bio/q2-books/intro.html) (33). These new mapping tools and databases, including Greengenes2, are powerful methods for partial 16S rRNA gene amplicon analysis. Regarding the high accuracy of analysis using the HOMD, the characteristics of the V1–V2 region of the 16S rRNA gene could contribute to this result. The greatest nucleotide heterogeneity and discriminatory power for the taxonomic analysis using the next generational sequencer has been reported for the V1–V2 regions (30, 34). Furthermore, the V1–V3 region, including the nucleotide information of the V1–V2 region, was recommended for the oral bacteria analysis when using the 97% similarity for OTU clustering (35). It was also reported that the V1–V3 region with Greengenes and HOMD showed a high resolution for the oral microbiome (22). We revealed a similar result the V1–V3 showed a relatively high resolution with HOMD, but not in Greengenes2. Meanwhile, according to recent reports conducting *in silico* analysis of the appropriate primer for the oral sample, the V3–V4, V4–V7, and V3–V7 regions showed a high percentage of read coverage for taxonomic assignment at the species level (36), and the V3–V7 region was recommended from the perspective of analysis accuracy, considering the number of internal genomes of 16S rRNA and the matching amplicon between different species (37). When targeting longer regions, such as the V3–V7 region, the long-read sequencer, such as Oxford Nanopore, might be the better option. It may also be the best option to select the primer targeting the V1–V2 region when using the HOMD and the short-read sequencer.

In addition, the selection of primer and database influenced the accuracy of not only the whole bacterial community but also individual bacterial species. Focusing on the analytical accuracy of *Streptococcus* in the mock1 community, with the 300 bp PE and the Greengenes2, the data obtained using the V1–V2, V3, V4, V4–V5, and V5–V6 primers showed the relatively higher accuracy than that of the V1–V3 and V6–V8 primers. *Bifidobacterium* showed lower analytical accuracy when the V3 primer was used compared with other primers for Greengenes2 (Fig. 5c). In the species-level analysis, the choice of primer has a stronger influence on the analytical accuracy than in the genus-level analysis. The analytical accuracy of *Streptococcus mutans* was relatively higher using the V3 primer than the others in the Greengenes2, but even when using the V3 primer, the analytical accuracy of *Streptococcus mutans* was relatively lower in SILVA than in Greengenes2 (Fig. 6c). A similar tendency was observed in both genus and species analyses in the mock2 community. At the genus-level analysis, the influence of primer and database seemed to be less than at the species level. Interestingly, the data obtained using the V1–V2 primer showed accurate results for all bacterial species in all databases except for the *Fusobacterium nucleatum* in the SILVA. It was suggested that the characteristics of the V1–V2 region, which provided the greatest nucleotide heterogeneity and taxon discriminatory power, strongly impact oral microbiome analysis. The Greengenes2 showed a relatively high resolution of each bacterial taxa compared with other databases in different primers, including the V3, V3–V4, V4, and V4–V5 at the species-level analysis. Focusing on *Corynebacterium* and *Cutibacterium*; the genus taxa and *Corynebacterium striatum* and *Cutibacterium acnes*; the species taxa of mock1 showed relatively low analytical accuracy in various primers, including the V3, V3–V4, V4, and V5–V6 primers with HOMD compared with the Greengenes2. These bacterial taxa do not primarily inhabit the oral cavity but are registered in the HOMD. This indicates that although the bacterial taxa are registered in the database used, their analytical accuracy is not necessarily high; each database has its characteristics suitable for the sample analyzed. These results imply that the accuracies of analysis for individual bacterial species are different based on the primer and database, and it influenced the analysis accuracy of comprehensive bacterial microbiota.

To our knowledge, this study is the first to investigate the characteristics of bacterial findings in the supragingival dental calculus in a Japanese population. The Shannon index value and the number of bacterial species detected with V1–V2 and HOMD at the species-level analysis were the highest, with 5.3 among all the primers and databases, and these results were consistent with the results of mock2 at the species level (Fig. 10a). It could be suggested that the combination of the HOMD with the V1–V2 primer has slightly higher discriminatory power for the oral-associated bacterial taxon than the SILVA and Greengenes2. We displayed the dominant bacterial genera in dental calculus including *Fusobacterium*, *Neisseria*, *Prevotella*, and *Streptococcus* (Fig. 9b). Previous reports showed that these bacterial genera were also dominated in the supragingival calculus using 16S rRNA amplicon sequencing analysis with V3–V4 primer and SILVA (38). The result of the PCA plots of the taxonomic and potential functional gene analyses showed a stronger influence of the individual differences in the sample itself than of the primer type in each database (Fig. 9c, 10c, and d). In this study, the dental calculus samples were collected from the subjects under the same treatment situation of supportive periodontal therapy, but the clinical situations differed among them; the age ranged from 46 to 83 years, the periodontal pocket depth ranged from 2.2 to 4.3 mm, and the sex consisted of 4 out of 5 males and 1 female. This might suggest the critical importance of controlling for clinical conditions to minimize the bias in the results obtained prior to sample collection. Although efforts have been made to avoid these sample biases, they are inevitable in clinical sample collection. However, the ANCOM-BC2 was published in 2023 and can detect the differentially abundant taxa among multi-groups, considering the covariates adjustments, including age and sex, to obtain more precise and accurate results (39). It is recommended to consider the bias hiding in each experimental step, not only for the selection of the sequencing length, primer, and database.

A limitation of this study is that the number of bacteria that constitute the mock2 was much lower than that of actual oral microbiota. However, it is difficult to imitate the oral microbiota because more than 700 species of bacteria can reside in the oral cavity, and half of these bacteria are difficult to culture. The number of dental calculus was 5, which was another limitation of this study to discuss the characteristics of the microbiota precisely. At present, the results of the mock analysis may aid in developing a more accurate analytic method. In the future, the development of mathematical models of oral microbiota can facilitate the simulation of the natural microbiota *in silico*.

In conclusion, this study suggested the combination of the stepwise trimming method, 300 bp PE, and V3-V4 or V4 primer with Greengenes2 and V1–V2 primer with HOMD for the analysis of oral microbiota using 16S rRNA gene amplicon analysis. This is the first comprehensive bacterial analysis of the supragingival dental calculus in Japanese subjects. The methods of this study will aid in setting appropriate sequence conditions for oral microbiome analysis as well as other environments.

## MATERIALS AND METHODS

### Preparation of mock communities

The overview of this experiment is displayed in Fig. 1. Two types of mock communities were used in this study. Mock1 community was DNA-Mock-001 (NBRC, Tokyo, Japan): a mixture of equivalent amounts of DNA from 15 species of bacteria obtained from various environments (https://www.nite.go.jp/data/000107974.pdf). Mock2 community was prepared in our laboratory and consisted of six bacterial species representatively associated with oral diseases (*Aggregatibacter actinomycetemcomitans* ATCC 43718, *Fusobacterium nucleatum* ATCC 25586, *Parvimonas micra* ATCC 33270, *Porphyromonas gingivalis* ATCC 33277, *Prevotella intermedia* ATCC 25611, and *Streptococcus sanguinis* ATCC 10556; Table S18). The following media were used to culture the bacteria: brain heart infusion broth (Becton, Dickinson and Company, Franklin Lakes, NJ, USA) for *A. actinomycetemcomitans* and *S. sanguinis*, brain heart infusion broth supplemented with hemin (5 mg/L) and vitamin K1 (1 mg/L) for *P. gingivalis* and *P. intermedia*, and fluid universal medium (40) for *F. nucleatum* and *P. micra*. Each species was cultured anaerobically at 37°C for 16–48 h. After the growth was confirmed, the bacterial DNA was extracted using the DNeasy PowerBiofilm Kit (Qiagen, Venlo, Netherlands) and RNase A (Takara, Tokyo, Japan) following the manufacturer’s instructions. DNA purity was determined using a Nanodrop One/One Microvolume UV-Vis Spectrophotometer (Thermo Fisher Scientific, Waltham, MA, USA), and the concentration was determined using a Quantus Fluorometer (Promega, Madison, WI, USA). The concentration of each sample was measured thrice, and an average value was calculated. Each bacterial DNA was diluted to 3 ng/µL and mixed into a single tube. The theoretical value of the bacterial abundance in the two mock communities was calculated using the formula: 16S rRNA gene copy number = [total genomic DNA (g) × unit conversion constant (bp/g)/genome size (bp)] × 16S rRNA gene copy number per genome (18). The genome size and 16S rRNA gene copy number were determined using the values listed in the American Type Culture Collection or referring to the National Center for Biotechnology Information and European Nucleotide Achieve databases. To confirm DNA contamination of other bacterial species not from the cultured bacterial species in the mock2 community, sequencing was performed for each DNA of six bacterial species using the V3–V4 primer (341F/806R: CCTACGGGNGGCWGCAG/GGACTACHVGGGTWTCTAAT), and the 300 bp paired-end (PE) reads obtained by the Miseq platform (Illumina, San Diego, CA, USA) were analyzed by QIIME2 and Greengenes2 (Table S19).

### DNA extraction from clinical samples

This study was performed in accordance with the Ethical Guidelines for Clinical Studies (2008 Notification Number 415 of the Ministry of Health, Labor, and Welfare) and was approved by the Ethics Committee of Tokyo Medical and Dental University (D2020-031).

A total of five Japanese patients with a history of periodontitis receiving supportive periodontal therapy every 3 to 6 months were included in this study. Clinical data, including age, sex, and average periodontal pocket depth for each subject, were collected (Table S20). The supragingival dental calculus samples were collected from the supragingival area before performing maintenance therapy using a sterile Gracey curette (Hu-Friedy, Chicago, IL, USA). Samples were stored at −20°C, and the DNA was extracted using the DNeasy PowerBiofilm Kit and RNase A. DNA purity and concentration were determined in similar methods as mock samples.

### Library preparation and Illumina sequencing of mock communities and clinical samples

The extracted DNA samples of mock1, mock2, and dental calculus samples were divided equally into nine aliquots for the independent amplification of nine regions of the 16S rRNA gene. The library preparations were performed for each aliquot using Ex Taq Hot Start Version (Takara, Tokyo, Japan) for the 1st PCR to amplify the target gene region and 2nd PCR to add the index to the edge of the amplicon, AMPure XP (Beckman Coulter Inc., Brea, CA, USA) for the purification of PCR amplicon, Qubit 3.0 Fluorometer (Thermo Fisher Scientific) for the measurement of DNA quantity, capillary electrophoresis with an Agilent 2100 bioanalyzer (Agilent Technologies, Santa Clara, CA, USA) for the evaluation of the length of PCR amplicon. The 1st and 2nd PCR conditions were 94°C for 2 min, followed by 20 cycles of 94°C for 30 sec, 50°C for 30 sec, 72°C for 30 sec, and finally 72°C for 5 min and 94°C for 2 min, followed by 8 cycles of 94°C for 30 sec, 60°C for 30 sec, and 72°C for 30 sec, and finally 72°C for 5 min, respectively.

The nine hypervariable regions and their primer sequences are listed in Table S21. Five samples were amplified with nine types of primers. The targeted hypervariable regions and sequences of primers were searched through Google Scholar using the search terms “miseq,” “16 s rrna gene,” and the name of the hypervariable region, such as “v1-v2.” The number of hits of hypervariable regions and sequences of primers were sorted from most to least, and a top of nine regions and primers used in at least 400 previous reports were included in this study. Each library replicated into three libraries and was sequenced on the MiSeq platform with 250 bp and 300 bp paired ends for the mock1 community and with 300 bp paired ends for both the mock2 community and dental calculus samples.

### Analysis of the sequenced data

Sequenced data were analyzed using QIIME2 (version 2022.2) (41), and the number and quality of raw reads were summarized using QIIME2 plug-in demux. The 1st PCR primer sequences for the library were removed using QIIME2 plug-in cutadapt, and the information from the trimmed reads was confirmed using demux. The DADA2 process was also performed to cut and merge the reads at appropriate locations according to the length and quality. The appropriate combinations of forward and reverse read lengths that left the highest number of reads after non-chimeric sequence removal were investigated. In the DADA2 process, the 250 bp PE reads of mock1 were cut at 15 locations between 90 and 230 bp, yielding 2,025 combinations, and the 300 bp PE reads of mock2 and dental calculus samples were cut at 20 locations between 90 and 280 bp, yielding 3,600 combinations. The parameter with the highest number of non-chimeric reads was used in the subsequent analysis. For the calculation of the length of the overlapped region, the following formula was used:

length of the amplified region (bp) = total length of the forward and reverse read after trimming (bp) – length of the overlap region (bp)

The taxonomic classification of amplicon sequence variants (ASVs) was performed using the qiime feature-classifier classify-sklearn based on the SILVA, Greengenes, Greengenes2, and HOMD. Silva 138 SSU Ref NR99 full-length sequences and Greengenes 13_8 99% OTUs full-length sequences were downloaded from the QIIME2 tutorial (https://docs.qiime2.org/2022.2/data-resources/). Taxonomic assignment using Greengenes2 was performed according to the tutorial (https://github.com/biocore/q2-greengenes2). The database referring HOMD used in this study was manually generated based on “HOMD_16S_rRNA_RefSeq_V15.22.fasta” and “HOMD_16S_rRNA_RefSeq_V15.22.qiime.taxonomy” from the HOMD website (https://www.homd.org/download#refseq). The bacterial names used as ideal data of the mock1 and mock2 communities were manually unified to the name in the SILVA. The bacteria not registered in the database and the inappropriate bacterial names, such as “metagenome,” were treated as “Unassigned” (Table S5 to S16). To obtain the theoretical read number of ASVs in mock communities, the mean, maximum, and minimum number of reads were calculated for each sample, and each number was multiplied by the relative value of bacterial species in mock communities and considered as the theoretical number of reads. The ASV abundance table was used to calculate the Shannon index for alpha diversity analysis. Then, the ASV abundance table was converted into a centered log-ratio (CLR) value using the R version 4.2.1 (2022-06-23) with the package compositions and zCompositions according to the CoDa_microbiome_tutorial (https://github.com/ggloor/CoDa_microbiome_tutorial) in downstream analysis. In the process, the ASVs with an average number of fewer than one read were removed from all the samples (42). The degree of analytical accuracy for each bacterial taxa comprising the mock communities was calculated based on the CLR value, and each value was averaged from three replicates. The CLR value, which ranged within ±5% error from the theoretical value, was symbolized as “+,” within ±5% to ±25% as “○,” and less than −25% or more than 25% as “-.” Bacteria not registered in the database were symbolized as “×.” The beta diversity analysis was performed with PCA using “prcomp” installed as a basic function of R to obtain the value of the principal component from the CLR values and visualized using the R packages “tidyverse” (43) and “ggplot” (44). The analysis of potential functional genes was performed using the q2-picrust plug-in (version 2021.11) based on the table and taxonomy data obtained from QIIME2 and Kyoto Encyclopedia of Genes and Genomes (KEGG) database (Tables S22 to S24) (45). The relative abundances of the potential functional genes were converted to CLR and analyzed using a scattered dot plot and PCA.

### Statistical analysis

The normality of data distribution converted to CLR was assessed using the Shapiro–Wilk test and Q-Q plot with the “shapiro.test” and “qqplot” in the R package “car.” Statistical tests using Dunne’s method were used for the α diversity based on the Shannon index. Microbiome similarity among different groups was tested using PERMANOVA “vegan::adonis2” (https://cran.r-project.org/web/packages/vegan/index.html, Sep. 1st, 2023). Spearman’s correlation coefficient and *P*-value were calculated based on the CLR values for the analysis of data similarity in each sample using the “corr.test” function in the R package “psych” (46).

## Data Availability

The data sets generated for this study can be found in the DNA Data Bank of Japan (DDBJ) with the following accession numbers for DNA sequencing: DRA016570 and DRA016571.

## AFTER EPUB

This article was published on 22 October 2024 with an error in the affiliations. The affiliations were corrected in the current version, posted on 29 October 2024.

## SUPPLEMENTAL MATERIAL

Figure S1 spectrum.03512-23-s0001.xlsx Survival rate of non-chimeric reads.

Figure S2 spectrum.03512-23-s0002.pdf Rarefaction curves.

Figure S3 spectrum.03512-23-s0003.pdf The top 10 bacterial species.

Table S1 to S4 spectrum.03512-23-s0004.xlsx Summary of the read processing and trimming length.

Table S5 to S16 spectrum.03512-23-s0005.xlsx Raw abundance data of sequence reads.

Table S17 spectrum.03512-23-s0006.xlsx Results of the similarity analysis with Greengenes using Spearman's correlation coefficient.

Table S18 to S19 spectrum.03512-23-s0007.xlsx List of bacterial species and raw sequenced reads in mock2.

Table S20 spectrum.03512-23-s0008.xlsx Sample metadata.

Table S21 spectrum.03512-23-s0009.xlsx List of the primer number and nucleotide sequence for each target region.

Table S22 to S24 spectrum.03512-23-s0010.xlsx Abundance of potential functional genes.

ASM does not own the copyrights to Supplemental Material that may be linked to, or accessed through, an article. The authors have granted ASM a non-exclusive, world-wide license to publish the Supplemental Material files. Please contact the corresponding author directly for reuse.
