# Supplementary material for: Optimal 16S rRNA gene amplicon sequencing analysis for oral microbiota to avoid the potential bias introduced by trimming length, primer, and database
Source: Microbiol Spectr. 2024 Oct 22;12(12):e03512-23. doi: 10.1128/spectrum.03512-23 (PMC11619299; doi:10.1128/spectrum.03512-23)
Supplement: Figure S2 — Rarefaction curves. [file spectrum.03512-23-s0002.pdf]

Supplementary Figure S2. Rarefaction curves of data using each primer and its replicates in mock1 with 250 bp and 300 bp PE and mock2 and the dental calculus samples with 300 bp PE.

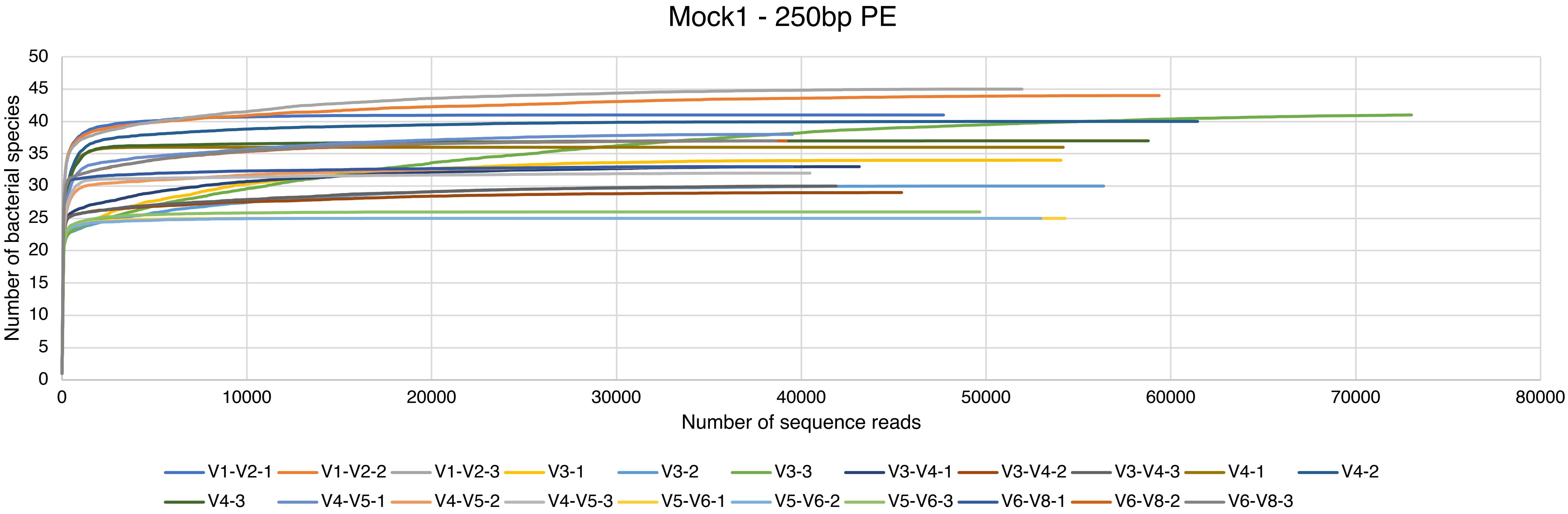

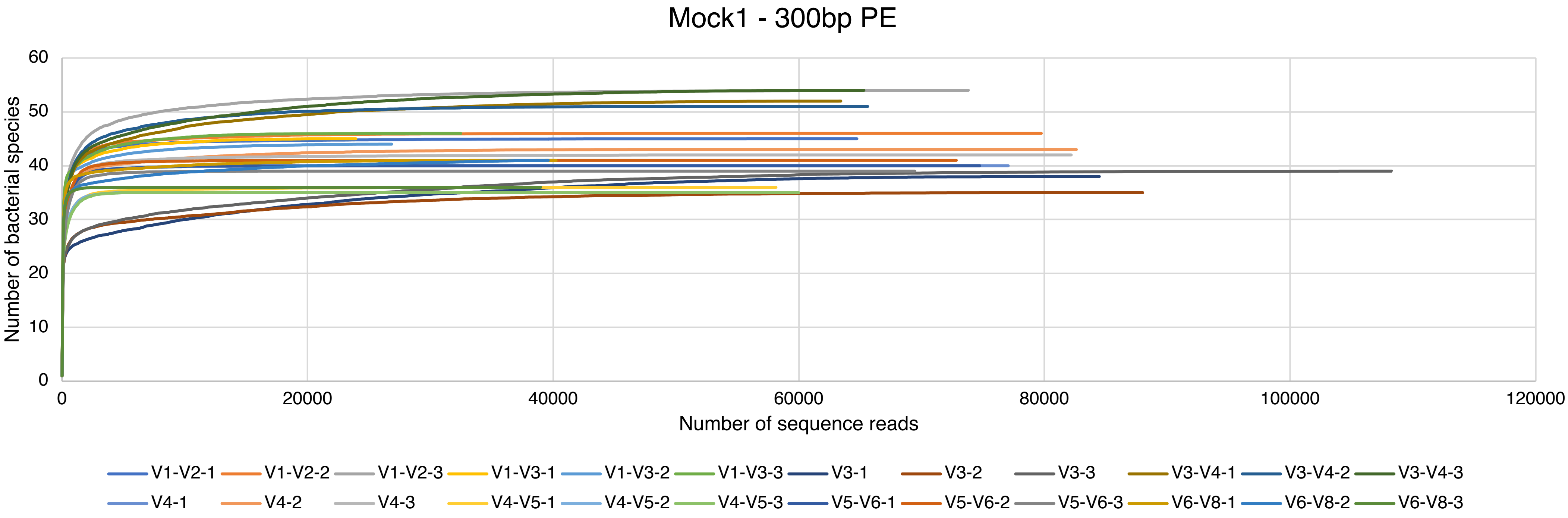

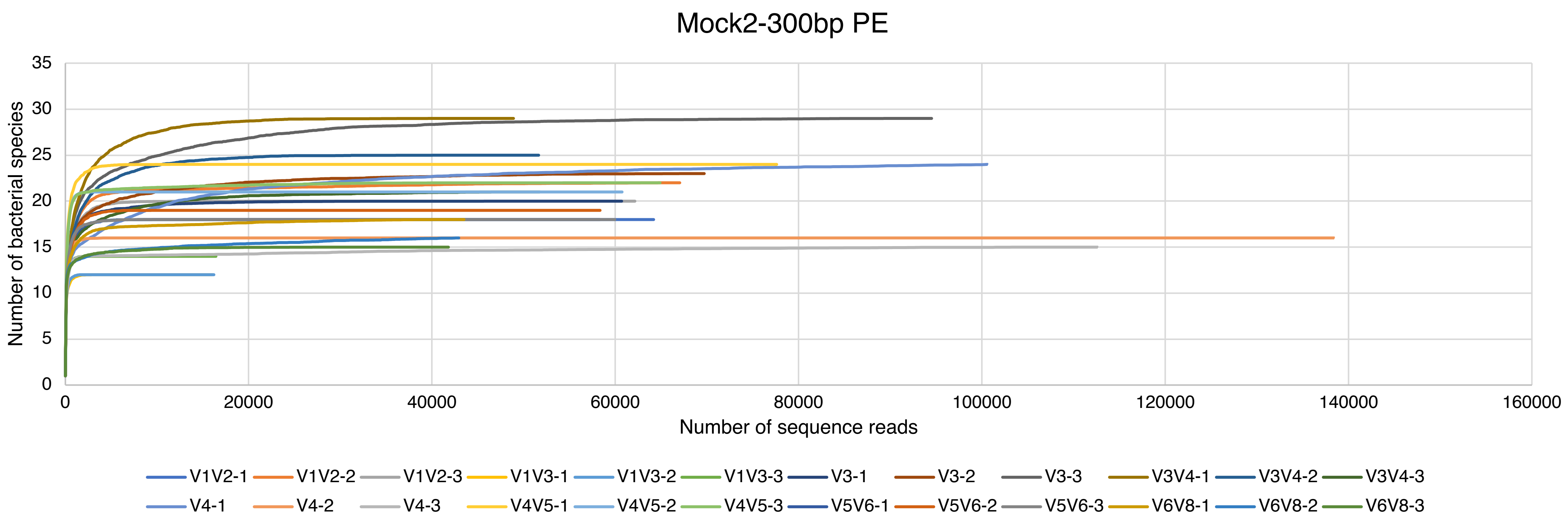

Mock2-300bp PE
Number of bacterial species
Number of sequence reads
0 5 10 15 20 25 30 35
0 20000 40000 60000 80000 100000 120000 140000 160000
V1V2-1 V1V2-2 V1V2-3 V1V3-1 V1V3-2 V1V3-3 V3-1 V3-2 V3-3 V3V4-1 V3V4-2 V3V4-3
V4-1 V4-2 V4-3 V4V5-1 V4V5-2 V4V5-3 V5V6-1 V5V6-2 V5V6-3 V6V8-1 V6V8-2 V6V8-3

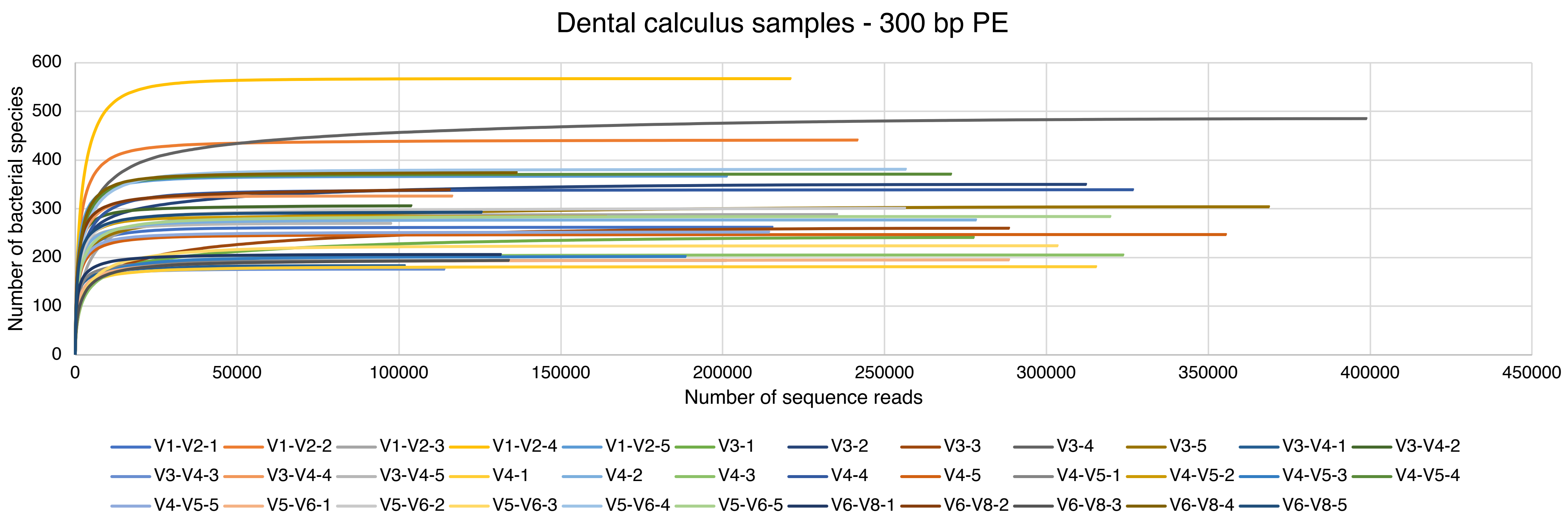

Dental calculus samples - 300 bp PE
Number of bacterial species
Number of sequence reads
0 100 200 300 400 500 600
0 50000 100000 150000 200000 250000 300000 350000 400000 450000
V1-V2-1 V1-V2-2 V1-V2-3 V1-V2-4 V1-V2-5 V3-1 V3-2 V3-3 V3-4 V3-5 V3-V4-1 V3-V4-2
V3-V4-3 V3-V4-4 V3-V4-5 V4-1 V4-2 V4-3 V4-4 V4-5 V4-V5-1 V4-V5-2 V4-V5-3 V4-V5-4
V4-V5-5 V5-V6-1 V5-V6-2 V5-V6-3 V5-V6-4 V5-V6-5 V6-V8-1 V6-V8-2 V6-V8-3 V6-V8-4 V6-V8-5
