# Supplementary material for: Optimal 16S rRNA gene amplicon sequencing analysis for oral microbiota to avoid the potential bias introduced by trimming length, primer, and database
Source: Microbiol Spectr. 2024 Oct 22;12(12):e03512-23. doi: 10.1128/spectrum.03512-23 (PMC11619299; doi:10.1128/spectrum.03512-23)

Supplementary Figure S3. The top 10 bacterial species based on CLR value at the genus level are shown in scattered dot plots using the primer targeting the V3–V4 region with Greengenes2 database and V1–V2 with HOMD.

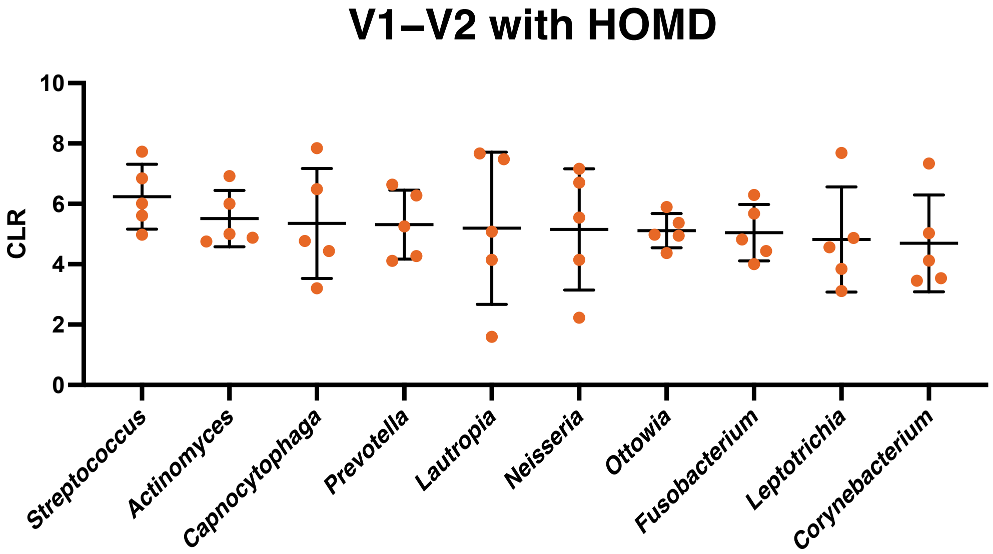

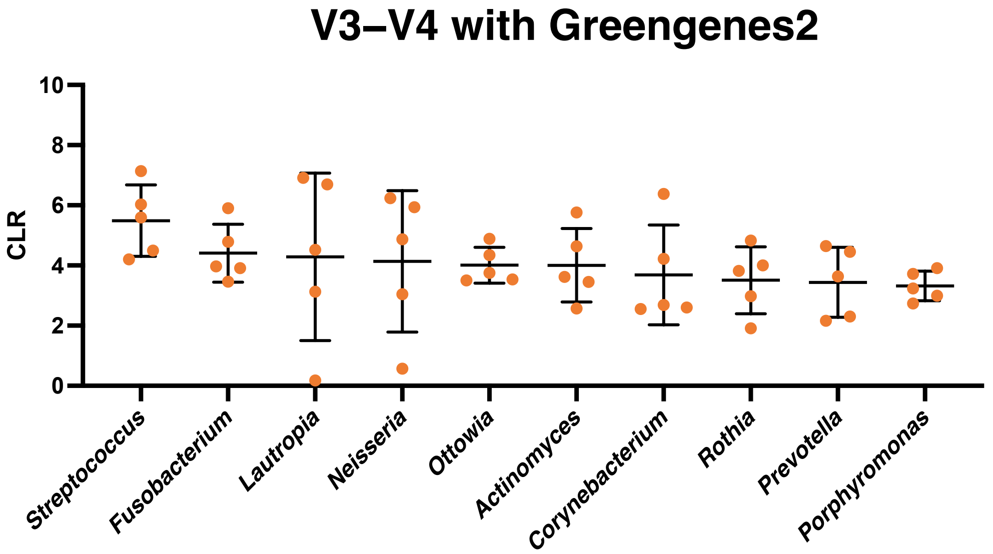

Supplement: Figure S3 — The top 10 bacterial species. [file spectrum.03512-23-s0003.pdf]
